# Rab1A Promotes Hepatic Steatosis by Suppressing Mitophagy via the Raf‐1/ERK1/2/PINK1 Signaling Axis

**DOI:** 10.1002/advs.202517520

**Published:** 2026-09-27

**Authors:** Li Zhang, Jianhua Li, Huilu Zhang, Qi Qin, Yichen Huang, Xiaofan Tian, Chao Sun, Binbin Li, Zhengxin Wang, Xin Zhang

**Affiliations:** ^1^ The Center for Bioinformatics and Computational Biology Shanghai Key Laboratory of Regulatory Biology Institute for Biomedical Sciences and School of Life Sciences East China Normal University Shanghai China; ^2^ Liver Transplantation Center Department of General Surgery Huashan Hospital & Institute of Organ Transplantation Fudan University Shanghai China; ^3^ Department of Digestive Diseases of Huashan Hospital Fudan University China; ^4^ Institute of Precision Medicine the Ninth People's Hospital Shanghai Jiao Tong University School of Medicine Shanghai China; ^5^ Ernest Mario School of Pharmacy Rutgers The State University of New Jersey Piscataway New Jersey USA; ^6^ Department of Pathology Shanghai Changzheng Hospital Navy Military Medical University Shanghai China; ^7^ Hongqiao International Institute of Medicine Tongren Hospital and Faculty of Basic Medicine Shanghai Jiao Tong University School of Medicine Shanghai China; ^8^ Digital Diagnosis and Treatment Innovation Center for Cancer Institute of Translational Medicine Shanghai Jiao Tong University Shanghai China; ^9^ Institute of Translational Medicine Shanghai Jiao Tong University (SJTU) National Center for Translational Medicine (Shanghai) SJTU Shanghai China

**Keywords:** lipid‐associated hepatocytes (LAHs), metabolic dysfunction‐associated steatotic liver disease (MASLD), mitophagy, Rab1A, Raf‐1/ERK1/2/PINK1 signaling

## Abstract

Despite extensive research, the etiology of metabolic dysfunction‐associated steatotic liver disease (MASLD) remains incompletely understood. Through analysis of single‐cell RNA sequencing (scRNA‐seq) data from murine and human MASLD models, we identify a lipid‐associated hepatocyte population and implicate Rab1A as its prominent player. Both global knockout and liver‐specific knockdown of Rab1A mitigate western diet‐induced hepatic steatosis in adult mice. We further show that knockdown of Rab1A in hepatic cells attenuates lipid accumulation by inducing excessive mitophagy. Mechanistically, Rab1A suppresses Raf‐1 activation, thereby inhibiting the MEK/ERK1/2 signaling cascade. Pharmacological inhibition of MEK/ERK1/2 via U0126 reverses lipid depletion and restores mitophagy attenuation in Rab1A‐deficient cells. We further demonstrate that ERK1/2 directly interacts with and phosphorylates PINK1 at Ser228, triggering PINK1‐Parkin‐dependent mitophagy. Critically, pharmacological activation of mitophagy by Urolithin A, or by C16‐PAF used as a research tool to engage the ERK1/2/PINK1‐Parkin axis, alleviates high‐fat diet‐induced hepatic steatosis and attenuates MASLD progression in mice. In human MASLD patients, Rab1A expression inversely correlates with activation of the Raf‐1/ERK1/2/PINK1 pathway. Collectively, our findings identify the Rab1A/Raf‐1/ERK1/2/PINK1 axis as a key regulator of mitophagy and MASLD pathogenesis, and highlight this pathway as a promising target for future therapeutic development.

## Graphical abstract

Why does fat accumulate in the liver? Our study reveals Rab1A as the molecular switch that silences the cell's mitochondrial cleanup crew. Turning Rab1A off reactivates mitophagy, clears excess fat, and reverses fatty liver disease in mice—offering a promising new therapeutic target for MASLD, a growing global health challenge.

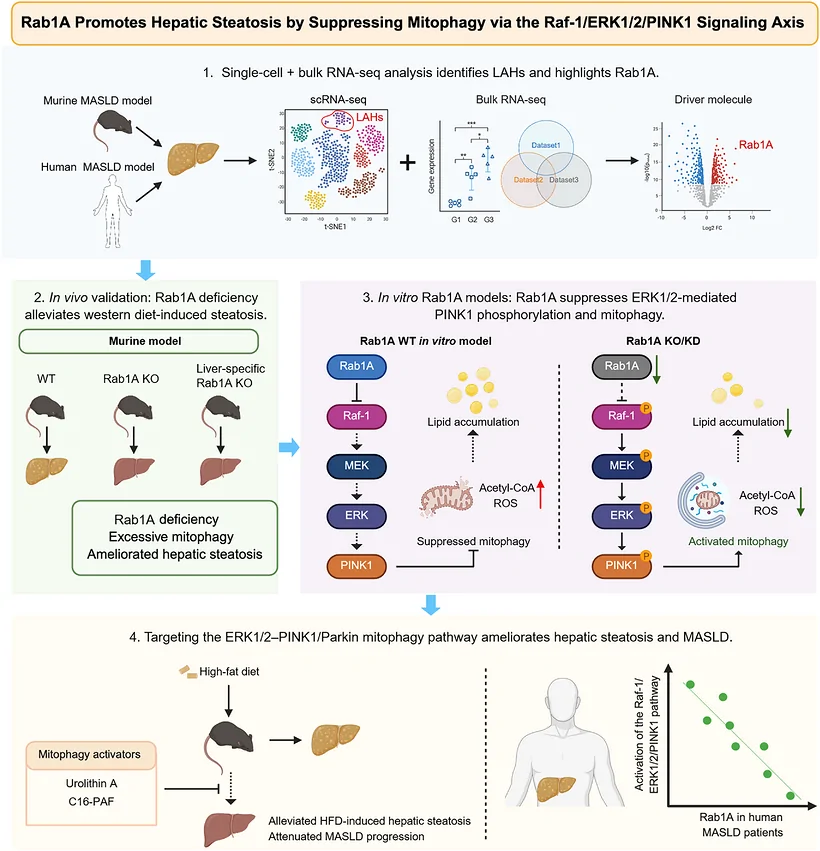

## 1 Introduction

Metabolic dysfunction‐associated steatotic liver disease (MASLD), one of the most representative chronic diseases, has been constituting a global problem as the Western pattern diet prevails [1, 2]. Characterized by accumulation of triglycerides (TGs) in the liver, hepatic steatosis is primed for metabolic dysfunction‐associated steatohepatitis (MASH), fibrosis, cirrhosis, and ultimately hepatocellular carcinoma, if left untreated [3]. The liver is a spatially heterogeneous organ, conducting diverse tasks by different subsets of hepatocytes [4]. The division of labor in the liver is known as liver zonation [5]. Periportal and pericentral hepatocytes exhibit clearly distinct metabolic features, profiled by their respective landmark genes. While the heterogeneity of the liver microenvironment plays a role in disease progression, the fundamental cellular mechanisms that drive initial lipid overload in hepatocytes remain incompletely understood. To halt the progression of MASLD, it is critical to uncover how the intracellular organelles responsible for lipid metabolism fail to maintain homeostasis during overnutrition.

Mitochondria are at the center of hepatic lipid metabolism, functioning as the primary site for fatty acid β‐oxidation [6]. Robust evidence indicates that mitochondrial dysfunction plays a pivotal role in MASLD and MASH pathogenesis [6, 7]. Under healthy conditions, mitochondrial quality is maintained by mitophagy, a selective autophagic process that clears damaged mitochondria. Numerous studies demonstrate that impaired mitophagy exacerbates obesity‐related metabolic diseases and MASLD [8, 9, 10, 11], whereas pharmacological activation of mitophagy (e.g., via Urolithin A) alleviates hepatic steatosis [12, 13, 14]. Despite its therapeutic importance, the upstream signaling networks that regulate mitophagy during high‐fat stress in MASLD remain elusive. It has been reported that two mitogen‐activated protein kinases (MAPKs) are required for mitophagy in Saccharomyces cerevisiae [15]. Besides, ERK1/2 signaling is involved in mitochondrial fission and aberrant mitochondrial morphology [16, 17]. In the liver, MAPK signaling exerts context‐dependent effects on lipid metabolism. Sustained activation of ERK1/2 and related MAPKs has been linked to enhanced lipid accumulation in hepatocytes and fatty liver [18, 19]. Conversely, ERK1/2 has been reported to promote hepatocyte autophagy and thereby attenuate liver steatosis by sequestering lipid droplets [20]. This duality indicates that the metabolic consequence of ERK1/2 activation depends on its downstream effectors and cellular context, raising the possibility that ERK1/2 engagement of the mitophagy machinery constitutes a protective arm of this pathway in the steatotic liver.

Rab1A is a small GTPase that is known for conducting intracellular vesicular transport between the cytosol and membranes [21, 22]. In line with this, Rab1A exerts its roles in the initiation of autophagy and endoplasmic reticulum (ER) complexity [23, 24]. It is worth noting that Rab1A belongs to the Ras superfamily, and it may function pleiotropically per se on account of GTPase activity [25]. Studies reveal that Rab1A regulates mTOR and NOTCH signaling pathways [26]. Rab1A activates mTORC1 in a Rag‐independent manner [27]. We then first studied the role of Rab1A in vivo employing a tamoxifen‐inducible knockout mouse model [28]. Amino acids‐Rab1A‐mTORC1 signaling controls insulin transcription and beta‐cell identity in adult mice. Beyond Rab1A, several Rab GTPases have emerged as regulators of hepatic lipid homeostasis. Rab7 serves as a central organizer of hepatocellular lipophagy, coordinating fusion of lipid droplet‐laden autophagosomes with lysosomes, and its inactivation impairs lipid droplet catabolism and aggravates steatosis [29]. Hepatic Rab24 was shown to suppress autophagic flux and mitochondrial connectivity, leading to hepatic steatosis and metabolic dysfunction [30]. Moreover, Rab2A promotes hepatic lipid accumulation downstream of AMPK–TBC1D1 by stabilizing PPARγ [31]. These findings position the Rab GTPase family at the crossroads of lipid trafficking, autophagy, and steatosis, yet the specific contribution of Rab1A to hepatocyte lipid handling has remained unexplored. Herein, we observed that Rab1A was strikingly upregulated in a subpopulation of lipid‐associated hepatocytes (LAHs) through our preliminary single‐cell RNA sequencing (scRNA‐seq) analyses of high‐fat diet (HFD)‐fed models. We further showed that Rab1A ablation improved hepatic steatosis and function by activating mitophagy via Raf‐1/ERK1/2/PINK1‐Parkin signaling under Western dietary feeding in adult mice.

## 2 Results

### 2.1 scRNA‐Seq Analyses Uncover Increased Rab1A Expression in Lipid‐Associated Hepatocytes

**FIGURE 1:**
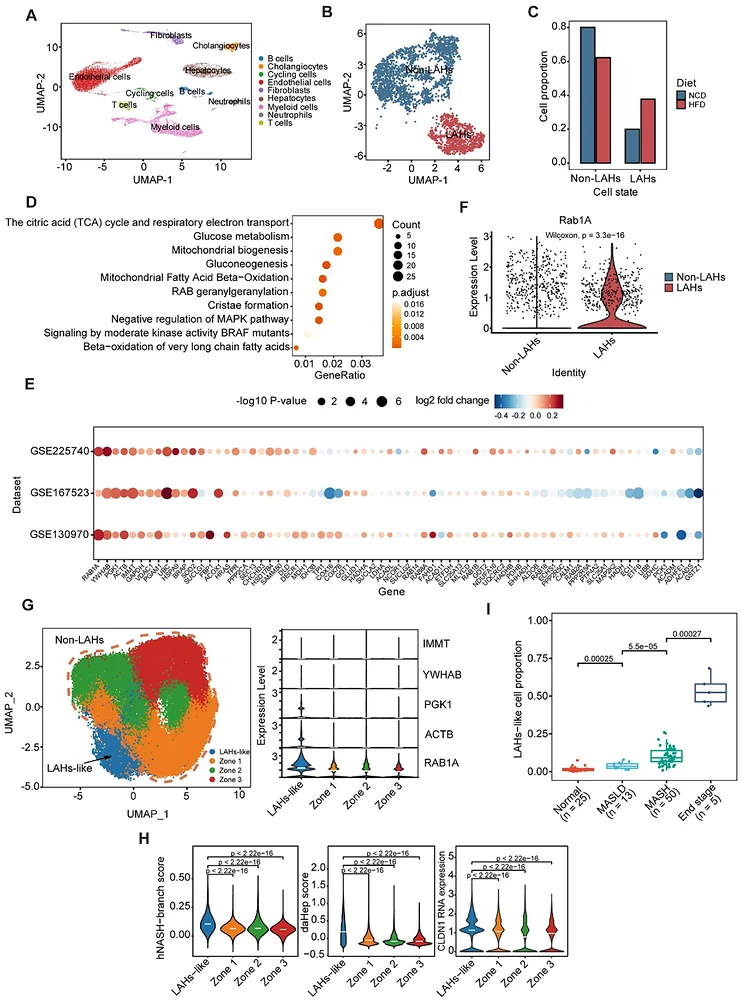
scRNA‐seq analyses uncover increased Rab1A expression in lipid‐associated hepatocytes. (A) UMAP showing cell annotation of scRNA‐seq dataset (GSE218299) from MASLD/MASH mouse model (NASH livers: *n* = 6, control livers: *n* = 6). (B) UMAP and unsupervised clustering of hepatocytes from (A). (C) Alterations in the proportion of non‐LAHs and LAHs on HFD. (D) GSEA showing the upregulated pathways of LAHs in a bubble plot. (E) Dot plot showing the top‐ranked marker genes of LAHs. Genes from the upregulated pathways were intersected with 3 bulk RNA‐seq datasets from MASLD patients (GSE225740: *n* = 93, GSE167523: *n* = 98, GSE130970: *n* = 78). (F) Violin plot showing the Rab1A expression in LAHs versus non‐LAHs. Analyzed by Wilcoxon test; *p*‐value was indicated. (G) UMAP showing cell annotation of snRNA‐seq dataset (GSE202379 and GSE289173) from patients with MASLD at different stages (Normal: *n* = 25, MASLD: *n *= 13, MASH: *n* = 50, end stage: *n* = 5). Markers of LAHs (*RAB1A, ACTB, PGK1, YWHAB and IMMT*) were verified in the LAHs‐like hepatocyte population. (H) Violin plots showing the distribution of signature scores for previously published disease‐associated hepatocyte populations (NASH‐branch2 and daHep), as well as the expression of *CLDN1*, across the four hepatocyte subpopulations. Statistical significance (*p* < 2.22e‐16) was assessed by the Wilcoxon rank‐sum test comparing LAHs‐like cells to each of the three zonated subpopulations. (I) Examination of LAHs‐like hepatocyte proportion in patients from different MASLD stages. Results are presented as mean ± SEM, analyzed by multiple Student's *t*‐tests, *p < 0.05, ****p < 0.0001.

To gain a close insight into the heterogeneity of hepatocytes in MASLD/MASH, we analyzed the scRNA‐seq data from a MASH mouse model on a Western diet [32]. Nine major hepatic cell types were identified as evidenced by respective marker gene expression (Figure 1A; Figure S1A). Further, hepatocytes were zoomed in and divided into two subsets based on the unsupervised clustering analysis (Figure 1B). Of particular interest, we noticed that the minor subpopulation of hepatocytes was remarkably increased on HFD (cell proportion from ∼0.2 to ∼0.4) whereas the major subpopulation displayed a slight decrease (cell proportion from ∼0.8 to ∼0.6, Figure 1C). Given the potential role of the minor subpopulation of hepatocytes in MASLD/MASH, we termed this subset LAHs, while the other majority subset was named non‐lipid‐associated hepatocytes (non‐LAHs).

Gene‐set enrichment analyses (GSEA) showed that genes involved in the tricarboxylic acid (TCA) cycle and respiratory electron transport, mitochondrial biogenesis, and gluconeogenesis were elevated in LAHs (Figure 1D), which was consistent with previous findings [33, 34]. By contrast, inflammation, lipoprotein assembly, and TP53‐related transcriptional regulation pathways were enriched in non‐LAHs (Figure S1B). To characterize the prominent players among these upregulated pathways in LAHs, three bulk RNA‐seq datasets using biopsies from MASLD/MASH patients were validated and crossed with genes of the GSEA bubble plot [35, 36, 37]. The top‐ranked five genes, that is, *RAB1A*, *YWHAB*, *PGK1*, *ACTB*, and *IMMT*, were set as markers for LAHs according to the upregulated expression levels in MASLD/MASH patients (Figure 1E; Figure S1C, Wilcoxon test, *P < 0.05*). Particularly, we noticed that Rab1A expression was strikingly increased in LAHs (Figure 1F). We then decided to probe the LAHs in liver biopsies from patients with MASLD/MASH. Two single‐nucleus RNA sequencing (snRNA‐seq) datasets using 93 people across the different stages of MASLD progression were analyzed [38]. Two subpopulations of hepatocytes emerged under unsupervised clustering analysis. Similarly, one subpopulation was in the majority, whereas the other subpopulation was minor (Figure 1G, left). Considering the human biopsies ranged from healthy to end‐stage disease, the complexity of the subpopulations of hepatocytes was presumably enhanced. Thus, the two subpopulations of hepatocytes were termed LAHs‐like hepatocytes and non‐LAHs, respectively. The non‐LAHs population could be further subdivided into Zone 1‐Zone 3 subclusters based on canonical liver zonation markers (Figure S1D), whereas LAHs‐like hepatocytes specifically expressed a panel of the above‐mentioned five LAHs signature genes (Figure S1E).

To compare the consistency between the LAHs cluster in mice and the LAHs‐like cluster in humans, a scoring approach using the differentially expressed genes (DEGs) from both clusters was set up. LAHs and non‐LAHs scores were then calculated by mapping homologous genes, respectively. We noticed that the LAHs score in LAHs‐like hepatocytes was higher than in non‐LAHs. Likewise, the non‐LAHs score was significantly elevated in non‐LAHs of human biopsies (Figure S1E). The inspection of the two populations of hepatocytes from humans further validated the analysis in mice. Moreover, three of the five markers (*PGK1*, *ACTB* and *RAB1A*) for LAHs from mouse models were significantly increased in LAHs‐like hepatocytes (Figure 1G, right, Figure S1F). Beyond our own analyses, the LAHs‐like population closely paralleled several independently reported MASLD/MASH‐associated hepatocyte subpopulations, notably disease‐associated hepatocytes (daHep) and hNASH‐branch2, both of which are characteristically expanded in MASH/NASH livers [39, 40]. In line with this resemblance, LAHs‐like cells displayed significantly higher signature scores for daHep and hNASH‐branch2 relative to non‐LAHs hepatocytes (Figure 1H). Additionally, the tight‐junction protein CLDN1, previously shown to be upregulated in MASLD and advanced liver fibrosis, was markedly enriched in LAHs‐like cells (Figure 1H). Collectively, these converging lines of evidence robustly define the LAHs‐like cluster as a conserved pathological hepatocyte state. Of note, the proportion of LAHs‐like hepatocytes was elevated as MASLD worsened, which was highest in end‐stage disease (Figure 1I). Subsequent differential expression analysis revealed a conserved transcriptomic signature: both murine LAHs and human LAHs‐like subsets were specifically enriched in adipogenesis‐related genes (e.g., *Ncor1, Plin2, Hnrnpu, Stat3, Ctnnb1*, and *Pck1*). In contrast, the non‐LAHs populations predominantly expressed genes associated with hepatocyte housekeeping and synthetic functions (e.g., *Alb, Apoa1, Cyp2e1*, and *Slc27a5*) (Figure S1G,H). Consistent with these expression profiles, module scores based on adipogenesis‐related gene sets indicated a significantly higher adipogenic capacity in both the LAHs and LAHs‐like subpopulations compared to their non‐LAHs counterparts (Figure S1I,J). These transcriptomic differences strongly support the functional heterogeneity of the two subpopulations. We further validated the role of Rab1A and the LAHs subpopulation during MASLD progression. qPCR analysis indicated that Rab1A mRNA levels in the liver tissues of MASLD patients were significantly elevated compared to healthy controls (Figure S1K). Consistently, Rab1A protein expression was also increased in MASLD liver tissues (Figure S1L). By employing Rab1A and PGK1 as markers to identify the LAHs subpopulation, we performed immunofluorescence staining on liver samples from healthy individuals and MASLD patients. The results demonstrated that the Rab1A^+^/PGK1^+^ LAHs population progressively expanded during MASLD progression and was spatially associated with regions of hepatic lipid accumulation (Figure S1M). Together, the scRNA/snRNA‐seq analyses unveiled a subpopulation of hepatocytes (termed LAHs) with marked expression of Rab1A, which increased as MASLD progressed.

### 2.2 Rab1A Deficiency Alleviates Western Diet‐Induced Hepatic Steatosis in Adult Mice

**FIGURE 2:**
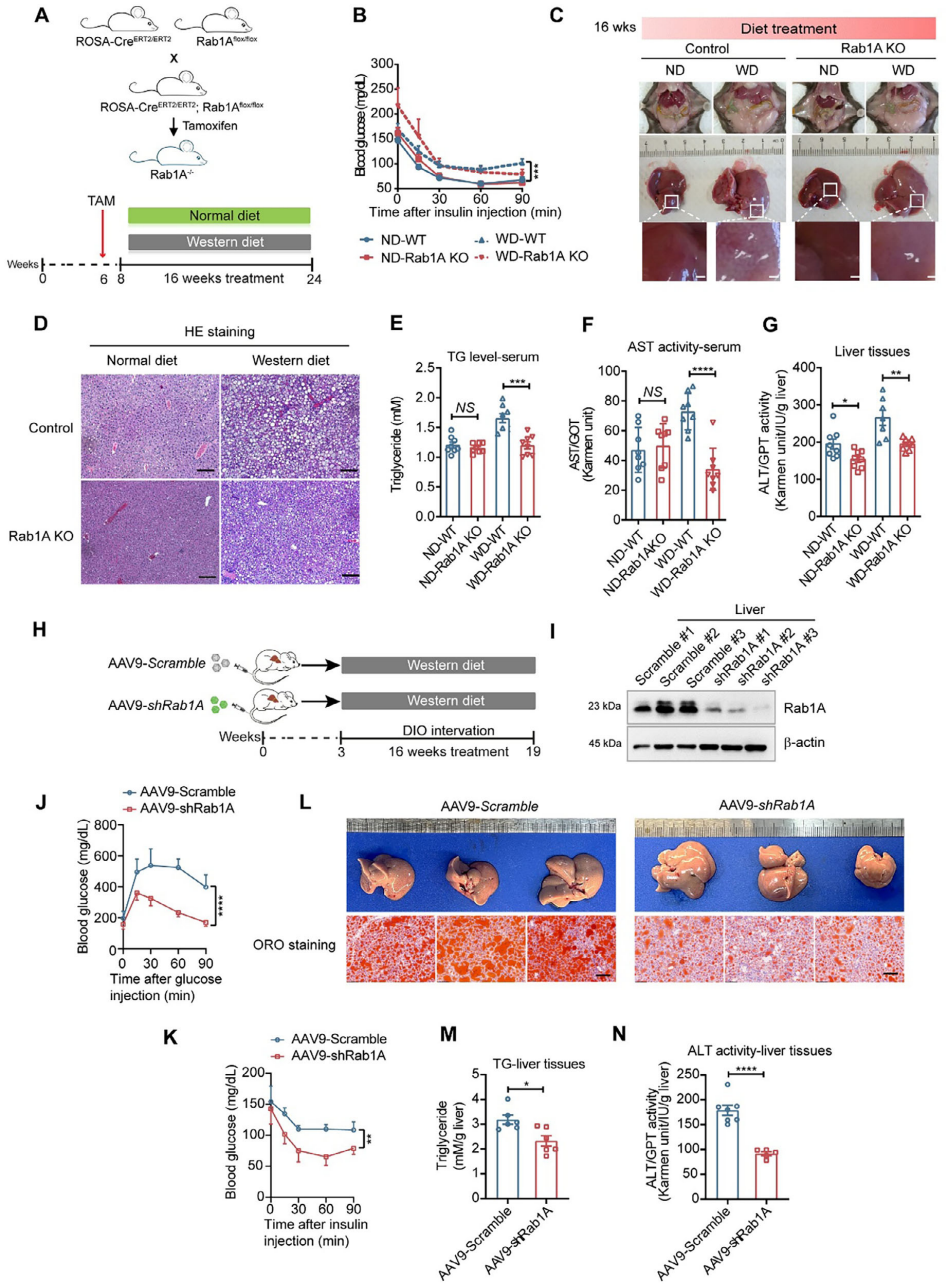
Rab1A deficiency alleviates western diet‐induced hepatic steatosis in adult mice. (A) Experimental scheme for establishing WD‐induced models with wild‐type control (WT) and Rab1A knockout (KO) mice (*n* = 8). (B) ITT of mice after 16 weeks on the diet. Results are presented as mean ± SEM (*n* = 8), ***p < 0.001, analyzed by two‐way ANOVA. (C) Representative images of livers in WT and Rab1A KO mice on the diet in situ. White speckles indicate lipid accumulation. Scale bar, 1 mm. (D) Representative H&E staining of liver sections in WT and Rab1A KO mice on the diet. White holes indicate hepatic steatosis. Scale bar, 50 µm. (E) Triglyceride levels in serum from 4 groups of mice were quantified using respective kits. Results are presented as mean ± SEM (*n* = 8). ***p < 0.001, unpaired Student's *t*‐test for two groups. (F) AST from serum of 4 groups of mice was quantified using the respective kits. Results are presented as mean ± SEM (*n* = 8). ****p < 0.0001, unpaired Student's t‐test for two groups. (G) Quantification of ALT in liver supernatants from WT and Rab1A KO mice on diet. Results are presented as mean ± SEM (*n* = 8). *p < 0.05, **p < 0.01, unpaired Student's *t*‐test. (H) Experimental design of WD‐induced model in Rab1A liver‐specific knockdown and control mice (*n* = 8). (I) Validation of Rab1A knockdown in liver upon AAV‐shRab1A delivery by immunoblot. (J,K) GTT and ITT performed with Scramble and Rab1A knockdown mice (16 weeks on diet). Results are presented as mean ± SEM (*n* = 7). **p < 0.01, ****p < 0.0001, analyzed by two‐way ANOVA. (L) Representative images of livers and ORO staining of liver sections with Scramble and Rab1A knockdown mice. Scale bar, 100 µm. (M) Quantification of triglyceride levels in liver supernatants from Scramble and Rab1A knockdown mice on a diet. Results are presented as mean ± SEM (*n* = 6). *p < 0.05, unpaired Student's *t*‐test. (N) Quantification of ALT in liver supernatants from Scramble and Rab1A knockdown mice on diet. Results are presented as mean ± SEM (Scramble = 7, shRab1A = 5). ****p < 0.0001, unpaired Student's *t*‐test.

Given that Rab1A was shown to be a prominent player in LAHs, its expression was increased in both mouse LAHs and human LAH‐like hepatocytes (Figure 1F; Figure S1D). To verify the potential role of Rab1A in MASLD, we used a tamoxifen‐inducible knockout mouse model since Rab1A knockout is embryonically lethal [28, 41]. Rab1A^Flox/Flox^ mice were crossed with R26‐CreERT2^+/−^ mice to allow inducible whole‐body knockout by tamoxifen (TAM). ROSA‐Cre^ERT2/+^; Rab1A^Flox/Flox^ and the littermate control mice were intraperitoneally injected with tamoxifen for 5 consecutive days at 6 weeks of age. To mimic a typical modern dietary pattern that is high in calories, we used either the Western diet (WD) containing 45% calories from fat or the normal diet (ND) containing 7% calories from fat for at least 16 weeks. Rab1A KO and WT mice were placed on WD or ND after verification of knockout efficiency by IHC in major organs (Figure 2A; Figure S2A). First, we noticed that WT mice on ND showed a modest increase in body weight gain compared to the Rab1A KO group on ND, while the difference in body weight gain between WT and Rab1A KO was more prominent on WD (Figure S2B,C). Since the HFD‐fed mouse is a well‐established model for studying type 2 diabetes mellitus (T2DM) [42], we next examined the fasting blood glucose, glucose tolerance, and insulin tolerance in our mouse model. WD‐fed WT mice demonstrated higher fasting blood glucose than the ND‐fed WT group, indicating the regimen worked in inducing diabetes‐like symptoms. Rab1A KO mice on ND showed persistently high fasting blood glucose compared to WT mice on ND, while Rab1A KO mice on WD displayed the highest fasting blood glucose among these groups (Figure S2D). WT mice on WD showed significantly worse glucose and insulin tolerance than their counterparts on ND, suggesting that the T2DM model was successfully established (Figure 2B; Figure S2E–G). Rab1A KO led to glucose intolerance on both WD and ND compared to WT littermates, while the glucose intolerance of Rab1A KO mice was aggravated under WD conditions (Figure S2F,G). Notably, ITT displayed a more rapid decrease in blood glucose level in Rab1A KO mice than in WT of both WD and ND groups, indicating that insulin sensitivity remained intact in Rab1A KO mice (Figure 2B). In our previous study, we have shown that Rab1A regulates insulin biosynthesis and β‐cell identity maintenance [28]. We next examined the islets and insulin expression on WD. Consistent with previous findings, the size of pancreatic islets was reduced, and insulin biosynthesis was impaired in Rab1A KO mice on WD and ND (Figure S2H,I). Therefore, it is highly likely that insulin insufficiency accounts for glucose intolerance in Rab1A KO mice on WD and ND.

Because the liver plays a central role in insulin sensitivity, we next examined the liver tissues from the four groups of mice at the end of diet treatment. Lipid accumulation was decreased in the livers of Rab1A KO mice compared with WT and was exacerbated on WD, as indicated by the size of lipid vacuoles in H&E staining (Figure 2C,D). The difference in lipid droplets between Rab1A KO and WT was reconfirmed by BODIPY‐488 staining using liver sections (Figure S2J). Liver weight gain of Rab1A KO mice on WD was remarkably decreased, although no difference was observed in terms of liver versus body weight ratio (liver/B.W.) (Figure S2K,L). Knockout of Rab1A improved the hepatic steatosis on WD, as evidenced by cholesterol and triglyceride levels (Figure 2E; Figure S2M,N). In line with this, impaired hepatic function was alleviated in Rab1A KO mice on WD compared with WT/WD as assessed by aspartate transaminase (AST) and alanine transaminase (ALT) activities (Figure 2F,G; Figure S2O). The fatty liver features were mitigated in WD‐induced Rab1A KO mice as indicated by histological NAFLD activity score (NAS) (Figure S2P).

To rule out the potential effects of other organs in Rab1A whole‐body KO mice, we generated a liver‐specific Rab1A knockdown mouse model. Liver‐specific knockdown of Rab1A was achieved using AAV9‐shRab1A under the control of the liver‐specific thyroxine‐binding globulin (TBG) promoter as described elsewhere [43, 44]. The mice were fed a WD for at least 16 weeks after validation of Rab1A knockdown efficiency by immunoblots (Figure 2H,I; Figure S2Q). Glucose and insulin tolerances were both significantly ameliorated in Rab1A liver‐specific knockdown mice (Figure 2J,K; Figure S2R,S). In parallel, lipid accumulation was decreased in Rab1A knockdown livers as indicated by Oil Red O (ORO) staining (Figure 2L). Likewise, liver weight gain of Rab1A liver‐specific KD mice on WD was dampened (Figure S2T). Moreover, Rab1A knockdown caused improved hepatic steatosis and function on WD as evaluated by triglyceride level, ALT activity, and NAS (Figure 2M,N; Figure S2U–W). Overall, these findings suggest that Rab1A ablation/knockdown alleviates hepatic steatosis, which in turn contributes to improved hepatic function.

### 2.3 Rab1A Knockdown Causes Excessive Mitophagy, Impaired De Novo Lipogenesis and Reduced Lipid Accumulation

**FIGURE 3:**
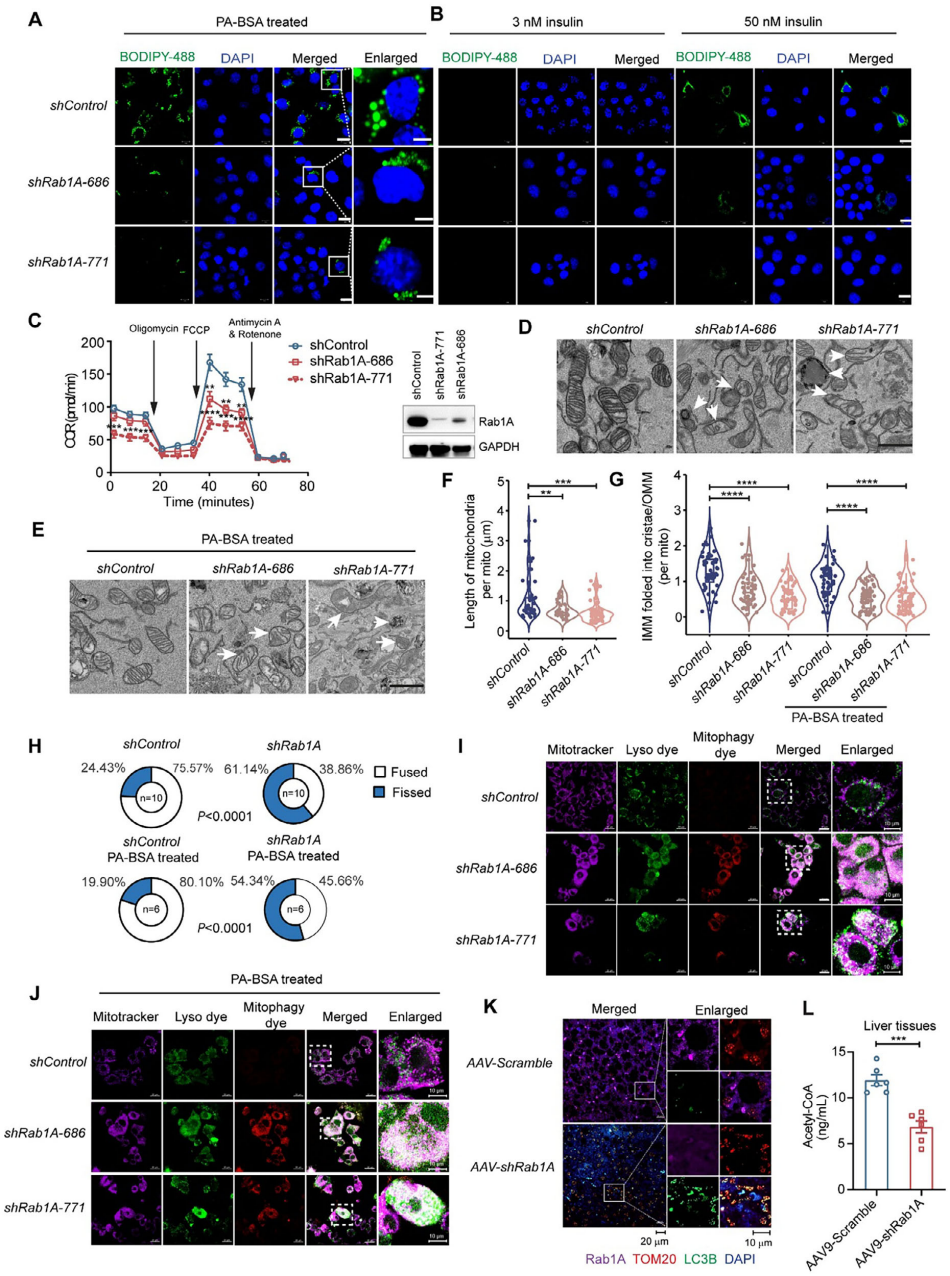
Knockdown of Rab1A induces excessive mitophagy and reduced lipid accumulation (A) Determination of lipid accumulation indicated by BODIPY‐488 in *shControl* and *shRab1A* AML12 cells treated with 50 µm PA‐BSA for 48 h. Scale bars: 10 and 5 µm. (B) Examination of lipid accumulation indicated by BODIPY‐488 in *shControl* and *shRab1A* AML12 cells treated with 3 and 50 nm insulin. Scale bar, 10 µm. (C) Assessment of mitochondrial function by OCR in *shControl* and *shRab1A* AML12 cells. Triplicate for each sample and repeated independently 3 times. Results are presented as mean ± SEM. **p < 0.01, ***p < 0.001, ****p < 0.0001, analyzed by multiple t tests using the Holm–Sidak method. (D,E) Representative high‐magnification electron micrographs of mitochondria in *shControl*, *shRab1A* AML12 cells (D) and treated with 50 µm PA‐BSA for 48 h (E). Arrows indicate impaired mitochondria and adjacent autophagosomes. Scale bar, 1 µm. (F and G) Quantification of mitochondrial length (F) and inner mitochondrial membrane (IMM) folded into cristae normalized to the outer mitochondrial membrane (OMM) perimeter of individual mitochondria (G) in *shControl* and *shRab1A* AML12 cells with normal complete media and 50 µm PA‐BSA for 48 h. At least 5 cells were analyzed for each group. Results are presented as mean ± SEM. **p < 0.01, ***p < 0.001, ****p < 0.0001, unpaired Student's *t*‐test. (H) Distribution of cells in (F) and (G) with fused (white) or punctate (blue) mitochondria. *n*, number of cells analyzed (part of the whole, chi‐square tests). (I) Evaluation of mitophagy by immunostaining of MitoTracker, Lyso, and Mitophagy dye in *shControl* and *shRab1A* AML12 cells. White speckles indicate occurrences of mitophagy. Scale bars: 20 and 10 µm. (J) Assessment of mitophagy by immunostaining of MitoTracker, Lyso, and Mitophagy dye in *shControl* and *shRab1A* AML12 cells treated with 50 µm PA‐BSA for 48 h. White speckles indicate occurrences of mitophagy. Scale bars: 20 and 10 µm. (K) Evaluation of mitophagy by co‐staining of TOM20 and LC3B in *Scramble* and *Rab1A* knockdown mouse liver tissues. Yellow dots indicate occurrences of mitophagy. Scale bars: 20 and 10 µm. (L) Acetyl‐CoA levels were detected using an ELISA kit in *AAV‐Scramble* and *AAV‐shRab1A* mouse liver tissues (*n* = 6). *** *p *< 0.001, unpaired Student's *t*‐test for two groups.

Following the findings of improved hepatic steatosis in Rab1A KO/knockdown mice, we next examined the role of Rab1A in *vitro*. A stable cell line expressing shRNAs against Rab1A was established in the murine hepatocyte cell line AML12. We observed considerable vacuoles in Rab1A knockdown cells (Figure S3A). The structure of the Golgi apparatus was not significantly altered in Rab1A knockdown cells, as assessed by GolgA2 staining (Figure S3B). Although Rab GTPases have been reported to play a role in unfolded protein response (UPR) and ER stress [45, 46], no remarkable difference was found in Rab1A knockdown cells as judged by ER‐stress markers (Figure S3C). To mimic the HFD‐challenged model in mice, AML12 cells were exposed to 50 µm BSA‐Palmitate saturated fatty acid complex (PA‐BSA) combined with DMEM/F12 complete medium for at least 48 h as described elsewhere [47, 48]. We further confirmed that the knockdown of Rab1A led to diminished lipid accumulation in PA‐BSA‐treated cells (Figure 3A; Figure S3D). The effect of insulin on DNL was assessed as well. High insulin concentration (50 nm) promoted DNL in AML12 cells, while Rab1A knockdown abolished the DNL triggered by insulin (Figure 3B; Figure S3E). These data were consistent with the role of Rab1A KO in *vivo* as shown in Figure 2. We then speculated that DNL‐related genes and signaling pathways may be impaired by Rab1A knockdown. Conversely, typical lipogenesis‐related genes were all greatly elevated in Rab1A knockdown cells as evaluated by qRT‐PCR (Figure S3F). Moreover, key molecules involved in lipogenesis such as ACC1, SREBP1, FASN, and SCD1 were not affected in Rab1A knockdown (Figure S3G). Of note, despite the significant upregulation of several lipogenic genes (e.g., *SREBP1*, *FASN*, and *SCD‐1*) at the mRNA level following Rab1A knockdown, their corresponding protein levels showed no significant alteration, a discrepancy likely attributable to post‐transcriptional and/or post‐translational regulatory mechanisms. Among these, lipolysis was remarkably reduced, as evidenced by immunoblotting of ATGL (Figure S3G). This reduction likely reflects homeostatic feedback regulation in response to the overall decrease in lipid accumulation observed in Rab1A‐deficient cells. These results suggest that Rab1A knockdown led to reduced lipid accumulation, which was not caused by attenuating DNL‐related gene/protein expression or lipid hydrolysis.

Mitochondria play a central role in DNL and lipid metabolism. We next examined the morphology of mitochondria in Rab1A knockdown cells by transmission electron microscopy (TEM). First, we noticed that mitochondria of Rab1A knockdown cells seemed to be smaller than those in *shControl* cells (Figure S3H). Meanwhile, we found that the number of mitochondria and mitochondrial DNA (mtDNA) were both reduced in Rab1A knockdown cells, suggesting decreased mitochondrial mass upon Rab1A knockdown (Figure S3I,J). Accordingly, mitochondrial respiration was severely dampened, as indicated by the lower oxygen consumption rate (OCR) levels in Rab1A knockdown cells (Figure 3C). TEM analysis of mitochondria from the stable cell lines exhibited that knockdown of Rab1A resulted in impaired ultrastructure and reduced cristae density, which were aggravated by PA‐BSA treatment (Figure 3D–G). We also noticed obvious autophagosomes near the mitochondria in Rab1A knockdown cells (shown by arrows in Figure 3D,E). Mitophagy is a process that involves removal of damaged mitochondria through autophagy [49]. Mitochondrial fission generates mitochondrial fragments and facilitates mitophagy [50, 51]. In accordance with this, Rab1A knockdown resulted in increased fissioned mitochondria under both normal and PA‐BSA‐treated conditions (Figure 3H; Figure S3K). Further, mitophagy was remarkably increased in Rab1A knockdown cells and exacerbated upon PA‐BSA treatment as evaluated by a Mitophagy Detection Kit (Figure 3I,J). Rab1A knockdown caused increased LC3B accompanied by fewer mitochondria (Figure S3L). Increased LC3B was concomitant with decreased lipid accumulation in PA‐BSA‐treated shRab1A cells (Figure S3M). We also observed enhanced hepatic mitophagy in liver‐specific Rab1A‐knockdown mice, accompanied by a concomitant reduction in lipid accumulation (Figure 3K). Besides, the amount of the activated form of PINK1(phosphorylated at S228; p‐PINK1 S228) and phospho‐Parkin (S65) were both elevated on Rab1A knockdown (Figure S3N), indicating that increased mitophagy occurred. Notably, we observed a significant upregulation of p62 levels in Rab1A‐deficient cells, coupled with a marginal reduction in the LC3‐II/I ratio (Figure S3N, right). This expression profile suggests that mitophagic flux may be impaired or obstructed following Rab1A knockdown. To further dissect how mitophagy attenuates lipid accumulation, we measured hepatic acetyl‐CoA levels and found a significant reduction in the liver tissues of Rab1A‐knockdown mice (Figure 3L). Given the dynamic nature of mitophagy, we next quantified intracellular acetyl‐CoA at 24, 48, and 72 h after PA‐BSA/DMEM treatment. A significant decrease was observed only at 72 h (Figure S3O), suggesting that mitophagy reaches a steady‐state level at this time point. As acetyl‐CoA is a direct substrate for de novo lipogenesis (DNL), its reduction would be expected to suppress DNL and thereby diminish lipid accumulation. In parallel, we assessed mitochondrial function under the same treatment conditions. Notably, at 72 h, Rab1A‐knockdown cells exhibited markedly elevated fatty acid oxidation (FAO) activity and reduced ROS levels compared with controls (Figure S3P,Q). These findings indicate that, despite a reduction in total mitochondrial mass, the functional capacity of the remaining mitochondria is substantially improved following Rab1A knockdown, thereby promoting clearance of excess free fatty acids and alleviating hepatocyte oxidative stress. In summary, Rab1A knockdown resulted in excessive mitophagy that elicited reduced mitochondria and reduced lipid accumulation.

### 2.4 Enhanced Raf‐1/MEK/ERK Signaling Activity Accounts for Triggering Mitophagy and Decreased Lipid Accumulation Upon Rab1A Knockdown

**FIGURE 4:**
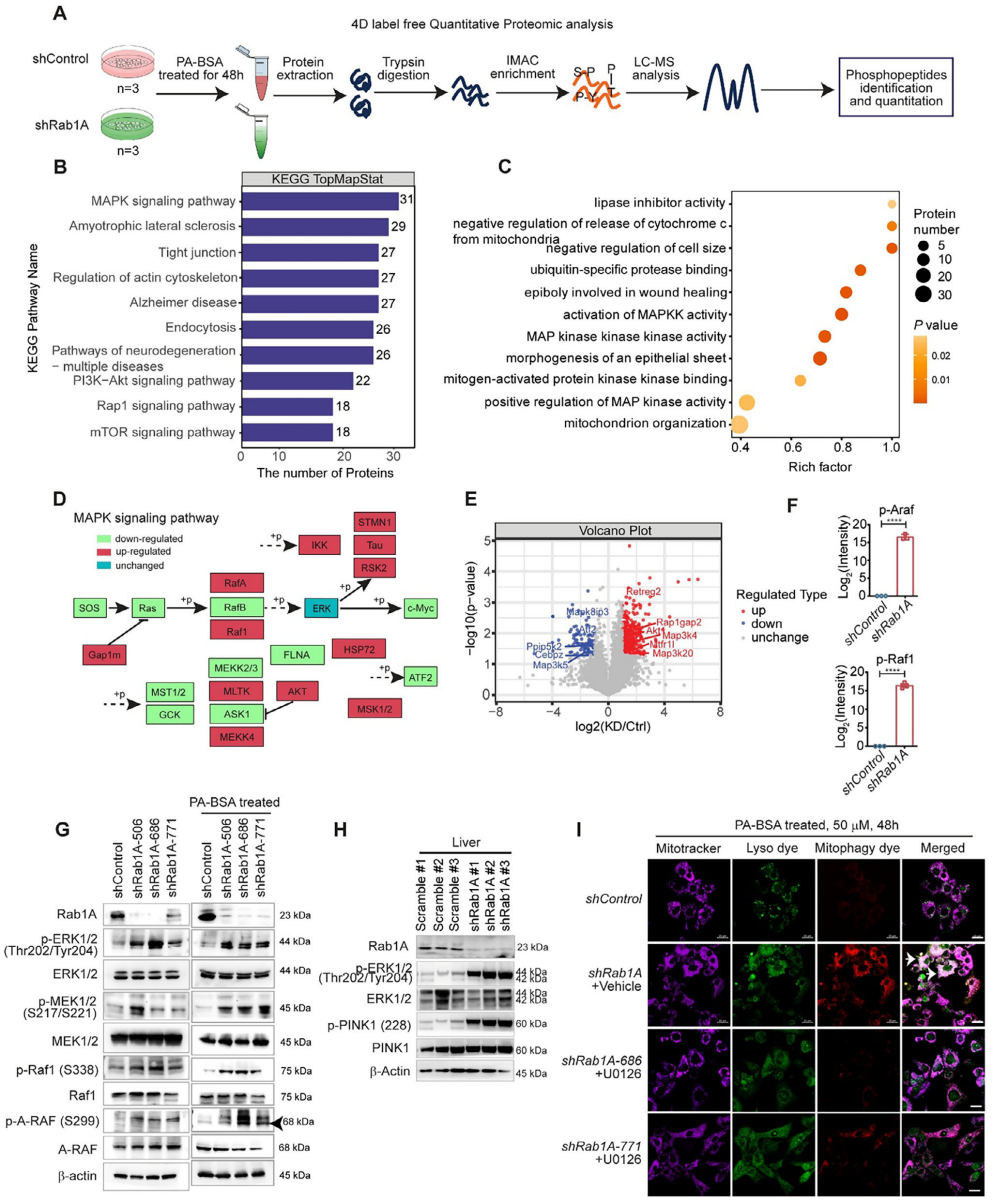
Enhanced Raf/MEK/ERK signaling axis accounts for triggering mitophagy and decreased lipid accumulation upon Rab1A knockdown (A) Flowchart scheme for 4D label‐free quantitative proteomic and phosphoproteomic analysis using *shControl* and *shRab1A* AML12 cells treated with 50 µm PA‐BSA for 48 h. (B) Top 10 enriched KEGG pathways of PA‐BSA‐treated Rab1A knockdown (KD) versus shControl cells, with differentially abundant proteins identified and analyzed by phosphoproteomic analysis. The lowest *p*‐value was selected for this analysis. (C) Differential abundance of phosphopeptides generated from (A) was shown in a bubble plot of potential signaling pathways. The size and color of each bubble represent the number of proteins in each pathway and the *p‐*value, respectively. (D) KEGG pathway highlighting the changed phosphorylated proteins in the MAPK signaling pathway extracted from both two cell types. Down‐regulated proteins are shown in green, up‐regulated in red color and unchanged in cyan. (E) Differentially expressed phosphopeptides between the two groups are shown by a volcano plot. *X‐*axis indicates the fold change (logarithmic conversion based on 2), and the *Y*‐axis indicates the *P‐*value (logarithmic conversion based on 10). Red dots represent up‐regulated phosphopeptides with significance, while blue dots indicate down‐regulated phosphopeptides with significance, and grey dots show the phosphopeptides without significance. (F) The intensities of phosphorylated A‐Raf and Raf‐1 are shown in both groups. Results are presented as mean ± SEM (*n* = 3), ****p < 0.0001, unpaired Student's *t‐*test. (G) Immunoblot analysis of components of the Raf‐1/MEK/ERK signaling pathway in *shControl* and *shRab1A* cells combined w/ or w/o PA‐BSA treatment. (H) Immunoblot analysis of phosphorylated ERK1/2 and PINK1 in *Scramble* and *shRab1A* mouse liver tissues. (I) Evaluation of mitophagy upon treatment with U0126 by immunostaining in *shControl* and *shRab1A* cells on PA‐BSA. White speckles indicated by arrows show occurrences of mitophagy. Scale bar, 20 µm.

To define the molecular basis of excessive mitophagy in Rab1A knockdown cells, we first detected the activation of the mTOR signaling pathway. Rab1A knockdown cells displayed no marked difference in terms of mTORC1 and AMPK signaling pathways as judged by phosphorylated S6K and AMPK (Figure S4A). Although activation of AKT at S473 was increased upon Rab1A knockdown, total AKT was elevated as well (Figure S4A). To gain more insight into the underlying mechanism, we next performed 4D label‐free quantitative phosphoproteomic analysis by LC–MS. Stable cell lines (i.e., *shControl* and *shRab1A*) were cultured in DMEM/F12 complete medium combined with PA‐BSA for 48 h, and then protein was extracted for further analysis (Figure 4A). Proteomic analysis validated the knockdown of Rab1A in the samples (Figure S4B). The MAPK signaling pathway was prominently changed between *shControl* and *shRab1A* cells, as suggested by phosphoproteomic analysis (Figure 4B,C). Players in the MAPK signaling pathway were primarily upregulated in Rab1A knockdown cells (Rab1A KD/Ctrl) (Figure 4D). Moreover, we noticed significant increases in MAP kinases (MAP3K phosphopeptides/proteins) in Rab1A knockdown (Figure 4E). Both phosphorylated A‐Raf and Raf‐1 were abundantly detected in Rab1A knockdown cells, while they were absent in *shControl* cells (Figure 4F). This was also congruent with the GSEA result in Figure 1D, depicting elevated mitochondrial biogenesis and negative regulation of the MAPK pathway in LAHs where Rab1A expression was significantly increased.

The MAPK/ERK pathway is a well‐known kinase cascade that mediates Ras activation [52]. Three major MAPK pathways have been defined so far: MAPK/ERK, SAPK/JNK and p38 MAPK [53]. The phosphoproteomic results were verified by immunoblotting. ERK1/2, but not JNK or p38 MAPK, was activated upon Rab1A knockdown. In line with this, MEK1/2, Raf‐1, and A‐RAF but not B‐RAF were activated in Rab1A knockdown cells as well (Figure 4G; Figure S4C). Notably, a significant elevation in the phosphorylation of ERK1/2 and PINK1 (Ser228) was also observed in liver tissues from Rab1A‐knockdown mice (Figure 4H). Intriguingly, U0126, an inhibitor of the MAPK pathway, strikingly rescued the phenotype of reduced lipid accumulation in Rab1A knockdown cells (Figure S4D). It is noteworthy that U0126 is an inhibitor of mitophagy as well. Rab1A knockdown cells treated with U0126 exhibited reduced mitophagy compared with vehicle control cells (Figure 4I; Figure S4E). Another mitophagy inhibitor Mdivi‐1, also restored lipid accumulation in Rab1A knockdown cells on PA‐BSA, suggesting that mitophagy accounts for DNL and lipid accumulation (Figure S4F). Together, these data strongly indicate that the elevated Raf‐1/MEK/ERK pathway is the fundamental cause of excessive mitophagy and reduced lipid accumulation in Rab1A knockdown cells.

### 2.5 Rab1A Constrains Raf‐1's Activation Through Direct Interaction

**FIGURE 5:**
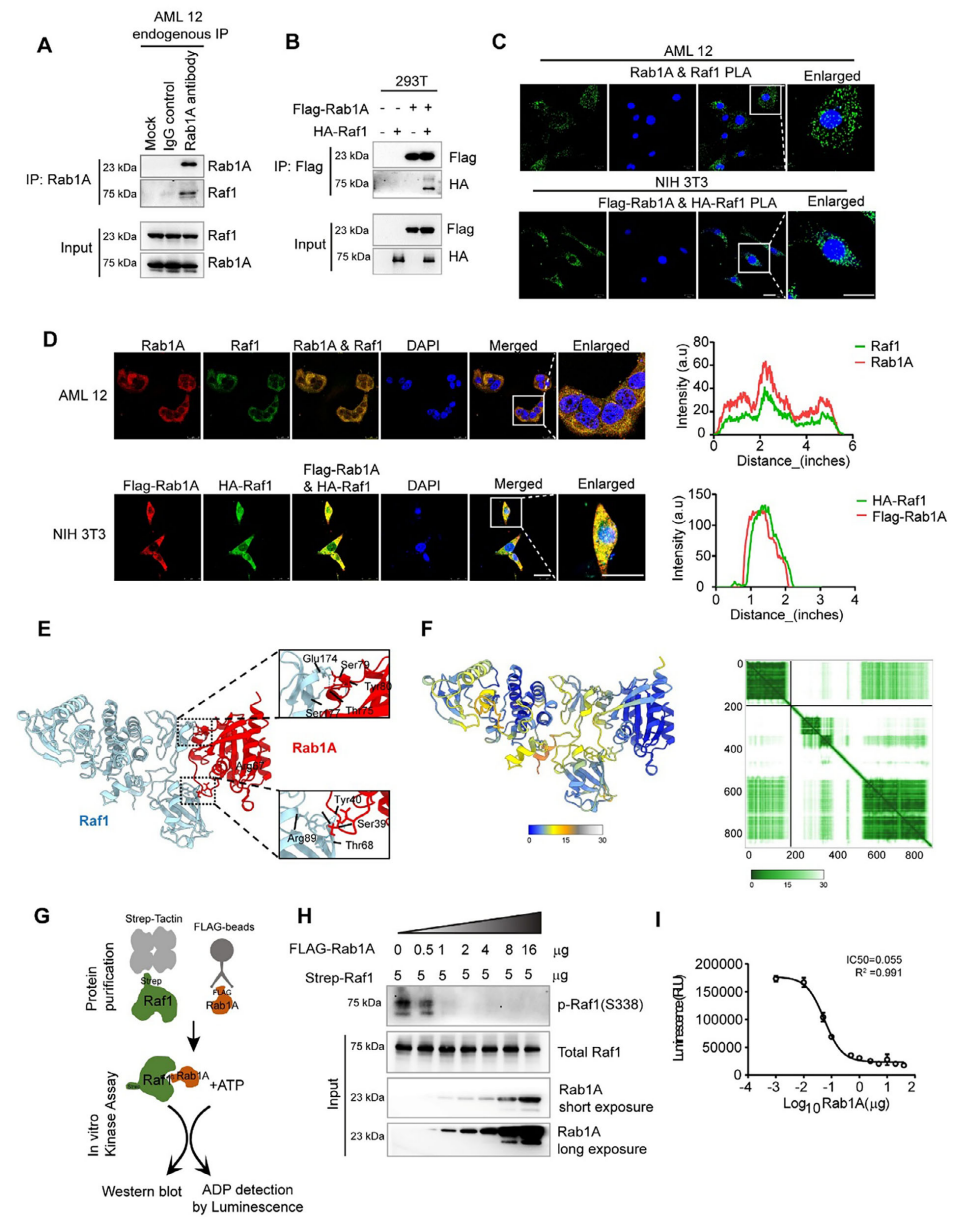
Rab1A constrains Raf‐1's activation through direct interaction. (A) Endogenous immunoprecipitation (IP) was performed using Rab1A antibodies in AML12 cells to verify the interaction between Rab1A and Raf‐1. (B) Exogenous IP was conducted using Flag beads. Flag‐Rab1A and HA‐Raf‐1 were co‐transfected at an equimolar ratio in 293T cells. (C) Interaction between Rab1A and Raf‐1 was demonstrated by proximity ligation assay (PLA) both endogenously and exogenously. Green fluorescent dots indicate the closeness of Rab1A and Raf‐1. Scale bars: 20 µm. (D) Representative confocal images show the colocalization of Rab1A and Raf‐1 both endogenously and exogenously. Fluorescence intensity was quantified using ImageJ to show the colocalization of the two proteins. Scale bars: 25 µm. (E) The three‐dimensional structure of Raf‐1 and Rab1A and potential interaction sites between these two proteins were predicted by the AlphaFold algorithm. Interaction sites are demonstrated by highlighting the key amino acids of both proteins. (F) pLDDT (predicted local distance difference test, left panel) and PAE (predicted Aligned Error, right panel) are shown for assessing the prediction results. (G) Experimental design for depicting the in vitro kinase assay. Strep‐Raf‐1 and Flag‐Rab1A were transfected into 293T cells and purified using respective beads per the guidelines. (H) Immunoblot analysis of the protein samples generated from the in vitro kinase assay. Phospho‐Raf‐1 (S338) and total Raf‐1 plus Rab1A were detected here. (I) Quantification of the suppressive effect on Raf‐1's phosphorylation using an ADP‐Glo kinase assay kit. Results are presented as mean ± SEM. IC50 was calculated using GraphPad Prism with a value of 0.055 µg.

We next asked why phosphorylated Raf‐1 and A‐Raf were considerably present in Rab1A knockdown cells and whether Rab1A regulates their activities. As Rab1A is a small GTPase that belongs to the Ras superfamily, it may affect the activity of Raf kinases. Because of the similarities of Raf‐1 and A‐Raf in protein structure, we focused on Raf‐1 modulated by Rab1A hereafter. Physical interaction between Rab1A and Raf‐1 was demonstrated by endogenous and exogenous immunoprecipitation (IP) (Figure 5A,B). The closeness of Rab1A and Raf‐1 was further manifested by proximity ligation assay (PLA) and immunofluorescence (IF) staining both endogenously and exogenously (Figure 5C,D). Protein–protein interaction (PPI) of these two proteins was assessed with the AlphaFold v2.3.1 algorithm. Although the interface of interaction was modest, they probably bind together, as indicated by values of predicted local distance difference test (pLDDT) and predicted aligned error (pAE) (Figure 5E,F). Further, the regulation of Raf‐1 activation by Rab1A was examined by employing an in vitro kinase assay. Raf‐1 activation was clearly inhibited by Rab1A with an IC50 of 0.055 µg as evaluated by immunoblotting and luminescent kinase assay (Figure 5G–I; Figure S5A).

Rab1A exists as either an activated (GTP‐bound) or inactivated (GDP‐bound) form owing to its intrinsic GTPase activity [54, 55]. We further examined whether Rab1A's GTPase activity per se plays a role in the activation of Raf‐1. Wild‐type Rab1A, its activated mutant Q70L (GTP‐restricted form), and the inactivated S25N (GDP‐restricted form) were co‐expressed with Raf‐1 in NIH 3T3 cells. The results showed that Raf‐1 mainly resided in the cytoplasm in Rab1A S25N‐expressing cells, while Raf‐1 was translocated into the nucleus at different levels in Rab1A wild‐type and Q70L‐expressing cells (Figure S5B). Additionally, the Rab1A S25N mutant immunoprecipitated less phosphorylated Raf‐1 than the wild‐type and Q70L mutant did (Figure S5C). Activation of the Raf‐1/MEK/ERK signaling module could be blocked by reconstituted Rab1A wild type and Q70L but not the S25N mutant (Figure S5D). Importantly, the reduced lipid accumulation in Rab1A knockdown cells was rescued by wild‐type Rab1A and the Q70L mutant, whereas the S25N mutant failed to do so (Figure S5E). Taken together, the data indicate that Rab1A constrains the activation of Raf‐1 through direct interaction in a GTPase‐dependent manner.

### 2.6 ERK1/2 Regulates PINK1's Phosphorylation via Interaction

**FIGURE 6:**
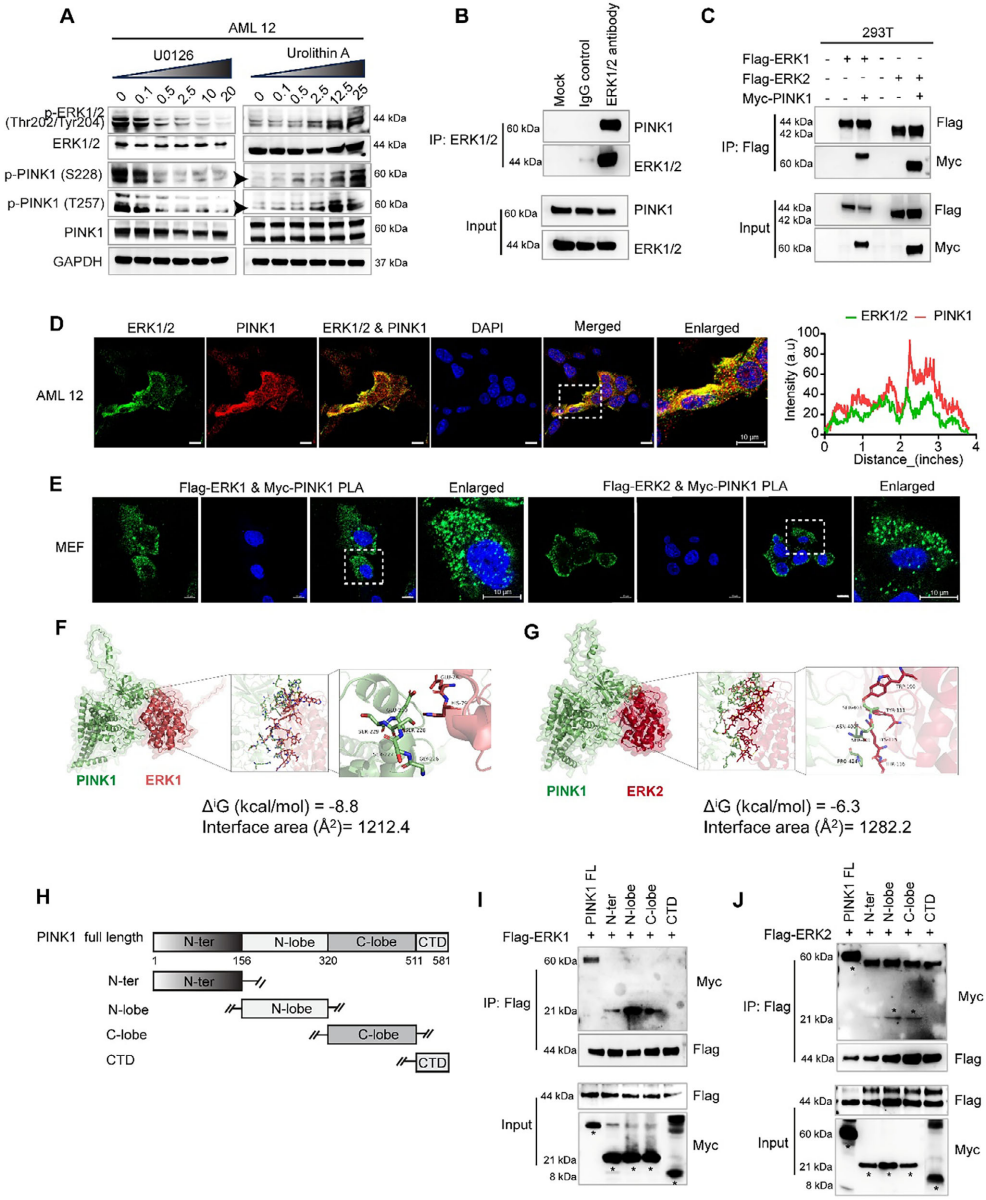
ERK1/2 directly regulates PINK1's phosphorylation via interaction. (A) Immunoblot analysis of phosphorylation of ERK1/2 and PINK1 upon treatment with gradient concentrations of U0126 and Urolithin A. (B) Endogenous immunoprecipitation (IP) was performed using ERK1/2 antibodies in AML12 cells to verify the interaction between ERK1/2 and PINK1. (C) Exogenous IP was conducted using Flag beads. Flag‐ERK1/2 and Myc‐PINK1 were co‐transfected at an equimolar ratio in 293T cells. (D) Representative confocal images show the colocalization of endogenous ERK1/2 and PINK1. Fluorescence intensity was quantified by ImageJ to show the colocalization of the two proteins. Scale bar, 10 µm. (E) Interaction between ERK1‐PINK1 and ERK2‐PINK1 was confirmed by proximity ligation assay (PLA) exogenously. Green fluorescent dots indicate the closeness of ERK1‐PINK1 and ERK2‐PINK1. Scale bar: 10 µm. (F,G) AlphaFold3‐predicted interaction between ERK1/2 and PINK1. Structural modeling of the ERK1/2–PINK1 complex with key interface residues highlighted. Binding energy and interface area are indicated. (H) Schematic illustration of functional modules of PINK1 and its truncated mutants. (I,J) Physical interaction regions between ERK1/2 and PINK1 were characterized by IP. Myc‐tagged truncated PINK1 mutants were co‐transfected with Flag‐ERK1 in (I) and Flag‐ERK2 in (J) and then subjected to IP using Flag beads. Inputs of PINK1 and its mutants were marked by asterisks.

The PINK1‐Parkin pathway plays a crucial role in mitophagy. As a mitochondrial damage sensor, PINK1 phosphorylates Parkin directly at S65 and initiates the removal of depolarized mitochondria [56]. The activation of PINK1 has been thought to be controlled by autophosphorylation at the residues S228, T257, and S402 [57]. Intriguingly, we noticed that both phospho‐PINK1 (S228) and phospho‐Parkin (S65) were both upregulated in Rab1A knockdown cells (Figures S3N,S4C,D). In addition, U0126 abolished the increased mitophagy caused by Rab1A knockdown (Figure 4I). Given that ERK1/2 was the prominent effector signaling pathway upon Rab1A knockdown, we thus speculated whether ERK1/2 directly regulates PINK1's phosphorylation. First, we noticed that the phosphorylation of PINK1 (T257 and S228) was gradually decreased with an increase of U0126 in a dose‐dependent manner (Figure 6A, left panel). Conversely, phosphorylation of PINK1 (T257 and S228) was increasingly elevated upon treatment with Urolithin A (Figure 6A, right panel). The phosphorylation of ERK1/2 was shown to have a concomitant tendency with that of PINK1. Next, the interaction between ERK1/2 and PINK1 was evidenced by endogenous and exogenous IP (Figure 6B,C). ERK1/2 and PINK1 displayed a subcellular co‐localized pattern indicated by IF staining both endogenously and exogenously (Figure 6D; Figure S6A). In addition, the spatial closeness of ERK1/2 and PINK1 was supported by PLA (Figure 6E; Figure S6B). The predicted interaction interfaces between ERK1/2 and PINK1 were obtained using the AlphaFold3 server. The two proteins interact through multiple sites. Representative sites of ERK1 & PINK1 and ERK2 & PINK1 are shown herein. The binding free energy (Δ^i^ *G*) between ERK1 and PINK1 is lower than that of ERK2 and PINK1, suggesting a stronger binding capacity of ERK1 & PINK1 (Figure 6F,G; Figure S6C,D). Of note, the key residue (S228) of PINK1 that is essential for its activation was included in the interaction interface of PINK1 & ERK1. We further characterized the physical interaction region of ERK1/2 and PINK1 by co‐IP. Four domain mutants, that is, N‐ter, N‐lobe, C‐lobe, and CTD, were constructed according to the functional modules of PINK1 as shown in the diagram (Figure 6H). Except for CTD, the other three domains interacted with ERK1 as shown by IP. Among these, the N‐lobe demonstrated the strongest binding capacity with ERK1, verifying the interaction interface of AlphaFold (Figure 6I). Comparatively, ERK2 displayed mild interactions with the N‐lobe and C‐lobe domains of PINK1, which was approximately consistent with the predictions by AlphaFold (Figure 6J). To further determine whether ERK1/2 regulates mitophagy via phosphorylation of the PINK1 S228 residue, we established stable PINK1‐knockdown AML12 cell lines and rescued them with either wild‐type PINK1 or its point mutants (S228A, phospho‐dead; S228E, phospho‐mimetic) (Figure S6E). Upon treatment with Urolithin A, we observed that p‐PINK1 (S228) levels were markedly lower in the PINK1^S228A^‐rescued cells compared to the other groups (Figure S6F). Furthermore, treatment with U0126 induced a dose‐dependent reduction in p‐PINK1 (S228) levels in cells expressing PINK1^WT^; however, this inhibitory effect was compromised in cells rescued with the PINK1^S228A^ mutant (Figure S6G). Moreover, while 10 µm Urolithin A successfully triggered robust mitophagy in PINK1^WT^ cells, this response was not observed in the PINK1^S228A^ reconstituted group (Figure S6H). To sum up, our data indicate that ERK1/2 directly interacts with PINK1 at Ser228, which probably leads to phosphorylation and activation of PINK1, initiating mitophagy.

### 2.7 Boosting Mitophagy via the ERK1/2/PINK1‐Parkin Signaling Axis Lessens Lipid Accumulation and Improves Western Diet‐Induced Hepatic Function

**FIGURE 7:**
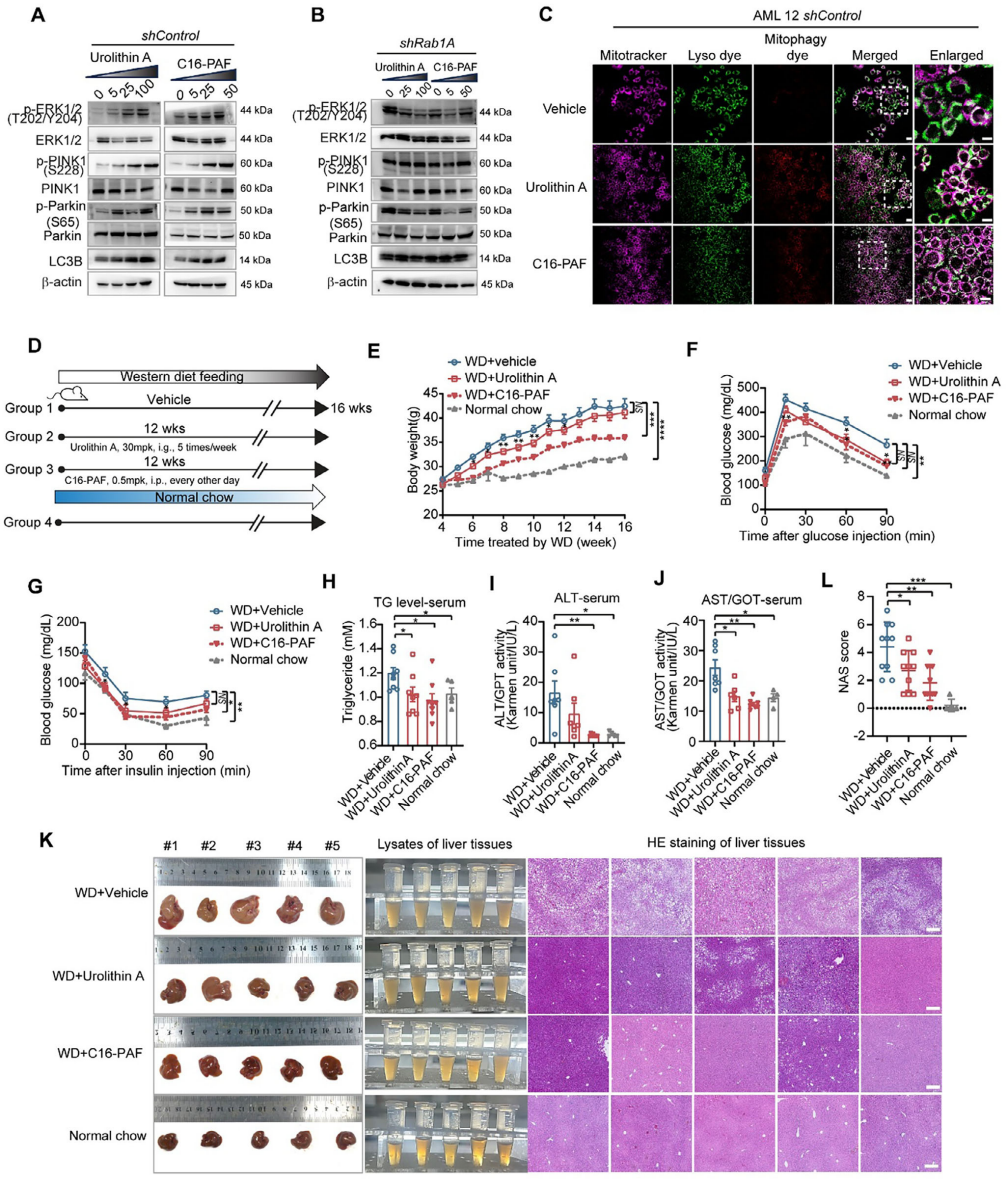
Boosting mitophagy via the ERK1/2/PINK1 signaling axis lessens lipid accumulation and ameliorates WD‐induced hepatic dysfunction. (A,B) Representative immunoblots performed with *shControl* and *shRab1A* cells treated with Urolithin A and C16‐PAF. The cells were treated for 24 h at the indicated concentrations. Phosphorylated ERK1/2 and mitophagy markers such as p‐PINK1 (S228), p‐Parkin (S65), and LC3B were detected. (C) Evaluation of mitophagy upon treatment with Urolithin A and C16‐PAF by immunostaining in *shControl* cells on PA‐BSA. White speckles indicate occurrences of mitophagy. Scale bars: 25 and 10 µm. (D) Experimental design for assessing the effects of two compounds (Urolithin A and C16‐PAF) in WD‐fed mice (for WD + Vehicle, WD + Urolithin A, WD + C16‐PAF, *n* = 10; for Normal chow, *n *= 5). (E) Weekly body weight of mice administered vehicle, Urolithin A, and C16‐PAF on diet, and normal chow‐fed mice. All results are presented as mean ± SEM (for WD + Vehicle, WD + Urolithin A, WD + C16‐PAF, *n* = 10; for Normal chow, *n* = 5). ***p < 0.001, ****p < 0.0001, analyzed by two‐way analysis of variance (ANOVA) followed by Šidák's multiple comparisons test. (F,G) GTT (F) and ITT (G) of 4 groups of mice after 16 weeks of treatment. All results are presented as mean ± SEM (GTT assay, WD + Vehicle and WD + Urolithin A groups *n* = 10, WD + C16‐PAF, *n* = 9; for Normal chow *n* = 5; ITT assay, WD + Vehicle, WD + Urolithin A, WD + C16‐PAF, *n *= 10; for Normal chow *n* = 5). *p < 0.05, **p < 0.01, analyzed by two‐way analysis of variance (ANOVA) followed by Šidák's multiple comparisons test. (H) Quantification of triglyceride levels in sera from 4 groups of mice. Results are presented as mean ± SEM (for WD + Vehicle, WD + Urolithin A, WD + C16‐PAF, *n* = 8; for Normal chow, *n* = 5). *p < 0.05, ** *p* < 0.01, unpaired Student's t‐test for two groups. (I,J) Quantification of ALT and AST in sera from 4 groups of mice. Results are presented as mean ± SEM (*n* = 7 in WD + Vehicle and WD + Urolithin A groups, *n* = 8 in WD + C16‐PAF, *n* = 5 in Normal chow for ALT) (*n* = 7 in WD + Vehicle, *n* = 6 in WD + Urolithin A, *n* = 8 in WD + C16‐PAF, *n* = 4 in Normal chow for AST). **p < 0.01, ***p < 0.001, ****p < 0.0001, unpaired Student's *t*‐test. (K) Multiple evaluations of hepatic steatosis with liver tissues from 4 groups. The overview of dissected livers (left panel) and supernatants of respective lysates (middle panel) and H&E staining analysis (right panel). The cloudier the supernatants, the more triglycerides the livers contain. Scale bar, 200 µm. (L) Pathological analysis performed with liver sections from 4 groups of mice. Severity of hepatic steatosis is indicated by NAFLD Activity Score (NAS). All results are presented as mean ± SD (for WD + Vehicle, WD + Urolithin A, WD + C16‐PAF, *n* = 10; for Normal chow, *n* = 5). **p < 0.01, ***p <0.001, unpaired Student's *t*‐test.

Based on the above findings, we hypothesized that boosting mitophagy by activating ERK1/2 signaling presumably ameliorates HFD‐induced hepatic steatosis. In fact, mitophagy modulation has been emerging as a novel therapeutic target in obesity‐associated cardiomyopathy [11]. Notably, the mitophagy activator Urolithin A exerts anti‐obesity effects in vivo [58]. C16‐PAF was selected from the compound library as it is also a potent MAPK and MEK/ERK activator. Urolithin A was used as a positive control herein (Figure S7A). We first examined the effects of both compounds in vitro. Both Urolithin A and C16‐PAF induced activation of the ERK1/2/PINK1‐Parkin signaling axis in a dose‐dependent manner, as indicated by phosphorylated PINK1 (S228) and Parkin(S65) in *shControl* but not Rab1A knockdown AML12 cells (Figure 7A). Given this high basal activation, the addition of the two compounds to *shRab1A* cells failed to induce a further increase in ERK1/2/PINK1 pathway signaling, which may trigger a negative feedback loop in *shRab1A* cells (Figure 7B). Particularly, C16‐PAF did not alter the activation of other MAPKs, such as p38 MAPK and JNK1/2 (Figure S7B).

We observed that the phosphorylated ERK1/2/PINK1‐Parkin signaling axis was substantially elevated upon Rab1A knockdown. Consistently, both Urolithin A and C16‐PAF treatments led to significant mitophagy in *shControl* cells (Figure 7C). In support of this, both compounds exhibited reduced mitochondria in a dose‐dependent manner in *shControl* cells, and decreased lipid accumulation was observed concomitantly (Figure S7C,D). In parallel, Rab1A knockdown cells displayed attenuated mitochondria and lipid droplets regardless of whether Urolithin A and C16‐PAF treatment was applied or not (Figure S7E).

The effects of Urolithin A and C16‐PAF in *vivo* were further explored using a WD‐fed model in WT mice (Figure 7D). During the period of treatment, C16‐PAF strikingly reduced body weight gain of mice on WD, while Urolithin A showed mildly decreased body weight (Figure 7E). Glucose intolerance on WD was improved by both compounds to a similar extent (Figure 7F; Figure S7F). Insulin sensitivity was significantly enhanced in C16‐PAF‐treated mice, and Urolithin A showed a modest improvement in insulin tolerance (Figure 7G; Figure S7G). Hepatic steatosis was ameliorated upon treatment with both compounds as assessed by total cholesterol (TC) and total triglyceride (TG) (Figure 7H; Figure S7H,I). Accordingly, the hepatic function of Urolithin A and C16‐PAF treated mice was improved as judged by alanine transaminase (ALT) and aspartate transaminase (AST) activities (Figure 7I,J; Figure S7J,K). The livers of Urolithin A‐ and C16‐PAF‐treated mice were smaller than those of vehicle control mice, with clearer supernatants of liver lysates (Figure 7K left and middle panels). Histological analyses demonstrated that lipid accumulation and steatosis were remarkably reduced upon treatment with both compounds (Figure 7K right panel and Figure S7L). Both Urolithin A and C16‐PAF improved the onset and progression of MASLD as indicated by the NAFLD activity score (NAS) (Figure 7L). Collectively, our results suggest that boosting mitophagy via ERK1/2/PINK1‐Parkin activation alleviates WD‐induced hepatic steatosis and dysfunction, demonstrating the robust role of C16‐PAF in MASLD.

### 2.8 Elevated Rab1A Correlates With Attenuated Activities of Raf‐1/ERK1/2/PINK1 in Patients With MASLD

**FIGURE 8:**
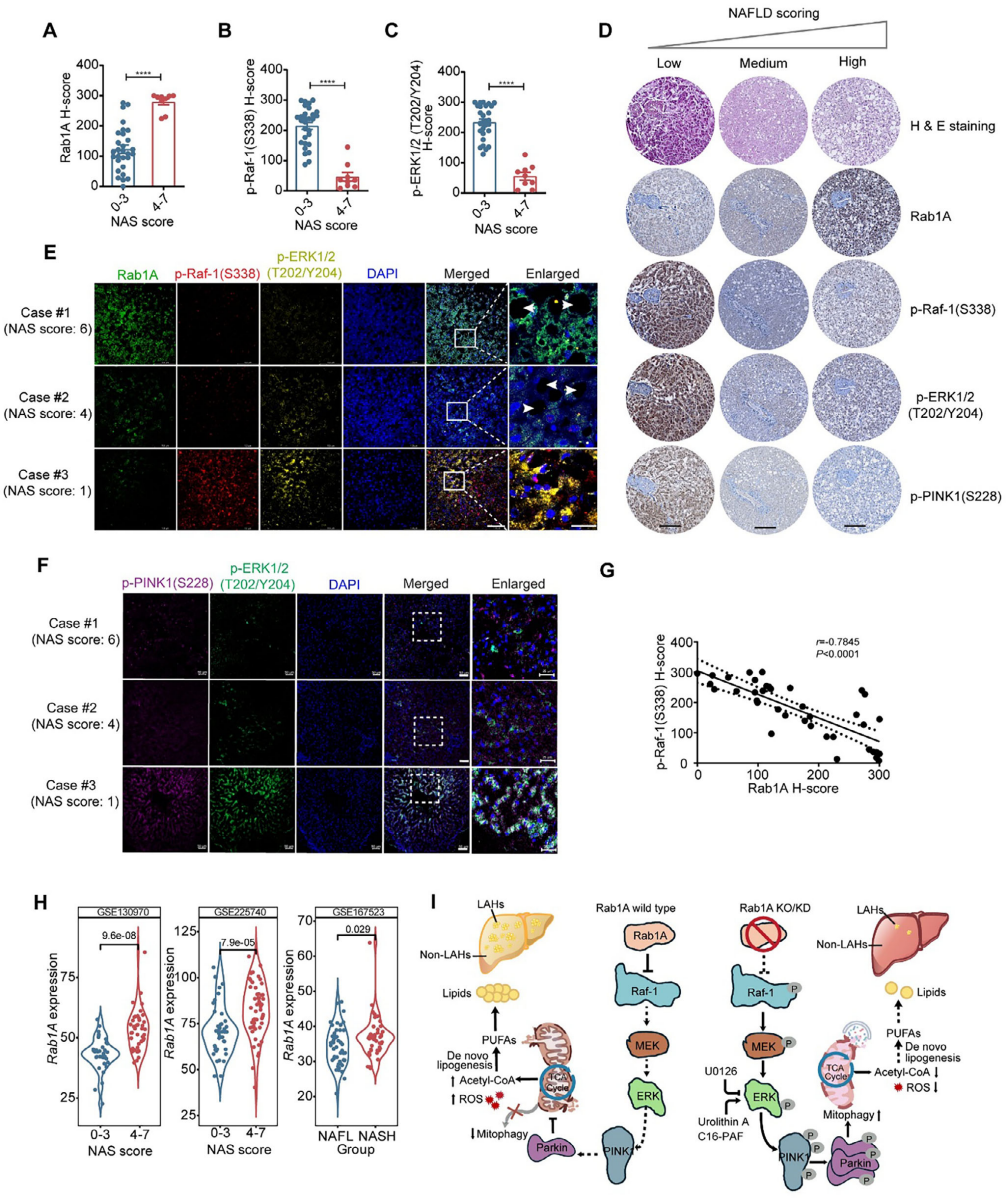
Elevated Rab1A correlates with attenuated activities of Raf‐1/ERK1/2/PINK1 in patients with MASLD (A) Analysis of Rab1A expression in human normal (NAS score 0–3) and MASLD (NAS score 4–7) liver tissues. All results are presented as mean ± SEM (*n* = 38). ****p < 0.0001, unpaired Student's *t*‐test. (B,C) Analyses of activated Raf‐1 and ERK1/2 by IHC staining with phospho‐specific antibodies against Raf‐1 (S338) and ERK1/2 (T202/Y204) in human normal and MASLD liver tissues. All results are presented as mean ± SEM (*n* = 38). ****p < 0.0001, unpaired Student's *t*‐test. (D) Trends of MASLD activity score (NAS), Rab1A expression, and activation of Raf‐1/ERK1/2/PINK1 analyzed by H&E staining and IHC with the indicated antibodies (Patient cohort = 38, as shown in Table S3). Representative images are shown here. Scale bar, 200 µm. (E) Examination of the negative correlation between Rab1A and activation of Raf‐1/ERK1/2 analyzed by IF staining with respective antibodies in high‐, medium‐, and low‐NAS‐score liver tissues. Representative images are shown here. Arrows indicate lipid droplets. Scale bars: 100 and 25 µm. (F) Concomitant activation of ERK1/2 and PINK1 was determined by IF staining using specific antibodies in high‐, medium‐, and low‐NAS‐score liver tissues. Scale bars: 50 and 25 µm. (G) Correlation plot showing Rab1A and p‐Raf‐1(S338) IHC staining (arbitrary units). Analyzed by a Pearson correlation test, the coefficient of correlation (*r*‐value) and the *p*‐value (*P*) are indicated, *R* ^2^: 0.6154. (H) Analyses of Rab1A expression in human normal and MASLD liver tissues using datasets GSE130970 (*n *= 78), GSE225740 (*n* = 93), and GSE167523 (*n* = 98) from the GEO database. Analyzed using the Wilcoxon test. *p*‐values (*P*) are indicated. (I) A working model depicting the role of Rab1A in mitophagy, DNL, and lipid accumulation in the liver. Rab1A constrains the activation of the Raf‐1/MEK/ERK1/2 cascade, which in turn inhibits mitophagy by decreasing p‐PINK1 (S228, T257) and p‐Parkin (S65). Overactivation of ERK1/2 by knocking out Rab1A or employing compounds (Urolithin A/C16‐PAF) causes excessive mitophagy. Enhanced mitophagy ultimately leads to decreased acetyl‐CoA, limiting substrate availability for de novo lipogenesis. On the other hand, mitophagy preserves and enhances the function of the remaining mitochondria, increasing FAO activity and reducing ROS levels, which together attenuate lipid accumulation and ameliorate MASLD pathology.

Given the potential impact of Rab1A on Raf‐1 and ERK1/2 in MASLD, next we verified the clinical relevance of the Rab1A/Raf‐1/ERK1/2 axis in patients with MASLD. We found that the expression of Rab1A was increased in MASLD patients with higher NAS scores (Figure 8A). Conversely, the activated forms of Raf‐1 and ERK1/2 were decreased in patients with MASLD, indicating that a suppressed Raf‐1/ERK1/2 module may be attributable to MASLD progression (Figure 8B,C). Patients with high MASLD scores were associated with elevated expression of Rab1A and reduced activation of Raf‐1, ERK1/2, and PINK1 (Figure 8D). In line with the above findings, the number of mitochondria was positively correlated with lipid accumulation in human liver tissues (Figure S8A). Further, mitophagy was remarkably augmented in liver tissues with low MASLD scoring as judged by TOM20 and LC3B co‐staining, and vice versa (Figure S8A,B). We then observed considerably higher expression of Rab1A and concomitantly lower phosphorylated Raf‐1/ERK1/2 in patients with higher MASLD scoring than in tissues with lower MASLD scoring (Figure 8E). Consistently, higher phosphorylated ERK1/2 co‐localized with higher phosphorylated PINK1 (S228) in patients with lower NAS score (Figure 8F). This tendency was confirmed by Pearson's correlation analyses as well. The expression of Rab1A was negatively associated with the activation of Raf‐1 and ERK1/2 as indicated by staining of phosphorylated‐form antibodies (Figure 8G; Figure S8C). The activation of ERK1/2 was significantly correlated with the activation of Raf‐1 (Figure S8D). Additionally, we analyzed 3 datasets of bulk RNA sequencing with MASLD/MASH cohorts from the GEO database [35, 36, 37]. The results showed that the expression of Rab1A was increased in patients with severe MASLD or MASH, which was congruent with our above findings. Together, these data indicate that the elevated expression of Rab1A correlates with the severity of MASLD, which attenuates the activation of the Raf‐1/ERK1/2/PINK1 module in patients with MASLD.

## 3 Discussion

scRNA‐seq allowed us to gain a sophisticated insight into the pathobiology of MASLD. In the current study, we identified an undescribed population of hepatocytes in MASLD/MASH liver biopsies from both mice and humans. Compared to the majority of other hepatocytes, the proportion of these cells was remarkably increased on HFD or as MASLD progressed. Thus, the population of hepatocytes was termed LAHs in mice or LAHs‐like hepatocytes in humans. It is well known that the liver is spatially heterogeneous in the form of zonation [4, 5]. Both Zone 3 and Zone 1 hepatocytes were reported to respond to HFD [59]. As hepatocytes are the cell type most affected by MASLD, our finding indicates that hepatocytes during MASLD progression could be divided into subpopulations at the single‐cell level, which is independent of spatial zonation.

We have been studying the role of Rab1A from the angle of intrinsic GTPase activity beyond vesicular trafficking. Rab1A was shown to operate through the ER/Golgi to activate mTORC1 and control glucose homeostasis by regulating insulin biosynthesis [27, 28]. In this study, we first noticed that *RAB1A* was the top‐ranked gene upregulated in LAHs/LAHs‐like hepatocytes. The function of Rab1A was further examined under a pathophysiological condition by employing an HFD‐induced model. Insulin tolerance is improved in mice with Rab1A deficiency. The ultimate reason for this phenotype lies in ameliorated hepatic steatosis upon Rab1A KO/knockdown. Subsequent mechanistic exploration uncovered that excessive mitophagy triggered by the upregulated Raf‐1/MEK/ERK1/2/PINK1‐Parkin signaling cascade accounts for reduced lipid accumulation in Rab1A knockdown cells. These findings reveal that Rab1A constrains the activation of Raf‐1/ERK1/2 and suppresses PINK1‐Parkin signaling, which leads to mitochondrial stasis that supplies excessive acetyl‐CoA for DNL in liver challenged with a WD regimen. These observations add Rab1A to a growing list of Rab GTPases that govern hepatic lipid homeostasis, although family members act through markedly divergent mechanisms. Rab7 serves as a central regulator of hepatocellular lipophagy, directing lipid droplets to lysosomes for degradation, such that its loss aggravates steatosis [29]. Intriguingly, hepatic Rab24 was shown to suppress autophagic flux and mitochondrial connectivity, leading to hepatic steatosis [30]. By contrast, Rab2A promotes hepatic lipid accumulation by stabilizing PPARγ downstream of the AMPK–TBC1D1 axis [31]. Against this backdrop, Rab1A is distinctive in that it influences lipid balance not through lipid‐droplet turnover but through mitochondrial quality control, restraining mitophagy via the Raf‐1/ERK1/2/PINK1‐Parkin cascade. This mechanistic distinction underscores the functional versatility of the Rab family in the fatty liver and identifies mitophagy as a previously unappreciated route by which a Rab GTPase shapes hepatic steatosis.

Mitophagy plays a pivotal role in multiple metabolic disorders, including MASLD, neurodegenerative diseases, obesity, and cardiovascular diseases [60]. Further, studies show that activation of mitophagy is beneficial in HFD‐induced obesity and cardiomyopathy [11, 61]. For example, compounds such as metformin and Urolithin A have been shown to protect against metabolic disorders by activating mitophagy [13, 62]. Yet, the molecular basis remains unclear. Earlier studies have shown that ERK/MAPK signaling regulates mitophagy and is required for mitophagy in *Saccharomyces cerevisiae* [15, 63]. We addressed the mechanistic knot of how ERK1/2 regulates mitophagy in the current study. Our data reveal that ERK1/2 regulates the phosphorylation of PINK1 at Ser228 by direct interaction. Phosphorylation of PINK1 is required for its activation and subsequent initiation of mitophagy. It has been thought that the activity of PINK1 is controlled by autophosphorylation at residues S228, T257, and S402 [57]. Intriguingly, we prove that both ERK1 and ERK2 physically interact with the N‐lobe (with residue S228 in it) of PINK1, which presumably leads to activation of PINK1‐Parkin signaling. Besides, our findings reconcile an apparent paradox: how enhanced mitophagy, despite reducing total mitochondrial mass, ameliorates hepatic lipid accumulation in MASLD. The resolution lies in the distinction between mitochondrial quantity and quality. By selectively eliminating damaged organelles, Rab1A knockdown engages two complementary outputs. First, the reduced mitochondrial pool diminishes cellular acetyl‐CoA, limiting substrate availability for de novo lipogenesis. Second, the residual mitochondria exhibit enhanced FAO activity and reduced Reactive Oxygen Species (ROS) generation, more efficiently disposing of excess free fatty acids while mitigating oxidative stress. The convergence of decreased lipogenic substrate supply and increased lipid catabolism explains the lipid‐lowering benefit of mitophagy and positions the Rab1A/Raf‐1/ERK1/2/PINK1 axis as a promising therapeutic target in MASLD (schematic working model in Figure 8I). Our data also indicate that Urolithin A promotes mitophagy through activating ERK1/2 and PINK1, uncovering the hidden signaling axis beneath Urolithin A. We also characterize C16‐PAF as a pharmacological tool to interrogate the ERK1/2‐PINK1‐Parkin axis in the context of hepatic steatosis.

The role of ERK1/2 in fatty liver has remained controversial, and our data should be placed in that context. Several studies associate sustained ERK1/2 and broader MAPK activation with enhanced lipid accumulation in hepatocytes and fatty liver [18, 19], whereas others report that ERK1/2 promotes hepatocyte autophagy and thereby attenuates steatosis [20, 64]. At least three variables may account for this divergence. First, the metabolic output of the cascade depends on which pool of ERK1/2 is engaged and which substrates it reaches. Nuclear ERK1/2 drives transcriptional programs that include lipogenic and proliferative targets, whereas the cytoplasmic and organelle‐proximal pool acts on local substrates such as PINK1 at the outer mitochondrial membrane [18]. The lipid‐lowering branch defined here belongs to the latter category and operates without a change in the transcriptional output of the pathway. Second, the amplitude and the duration of ERK1/2 activation differ substantially between experimental settings. In our model, the pathway is engaged selectively downstream of Raf‐1 and A‐Raf, and its amplitude is set by the loss of a single restraint, Rab1A, which is a different regime from pharmacological or oncogenic pathway hyperactivation. Third, part of the evidence for a lipogenic role of ERK1/2 derives from systemic MEK inhibition or from global genetic models, in which responses of adipose tissue, myeloid cells and hepatic stellate cells are superimposed on the hepatocyte‐autonomous response, and the disease stage under study, simple steatosis or established steatohepatitis with inflammation and fibrosis, is a further source of variation [65]. We therefore do not regard ERK1/2 activation as uniformly protective or deleterious in the fatty liver. What our data support is a defined, substrate‐specified arm of the cascade, Raf‐1/MEK/ERK1/2/PINK1‐Ser228, whose engagement lowers hepatic lipid content, and the net metabolic consequence of ERK1/2 activation is expected to depend on which of its downstream branches predominates in a given context.

Raf kinases are activated by membrane‐localized Ras through a high‐affinity interaction between Ras and Raf's Ras‐binding domain (RBD), which in turn results in activation of the RAF/MEK/ERK cascade involving a variety of cellular processes [66, 67]. Our data show that Rab1A suppresses the activation of Raf‐1 and A‐Raf but not B‐Raf. This distinct regulation of Raf activation is markedly reminiscent of Rap1, a small GTPase that is very similar to Ras. Rap1 is a selective activator of B‐Raf and an inhibitor of Raf‐1 [68, 69]. Moreover, other small GTPases such as Rho and Rac1 are reported to be involved in Raf activation as well [70, 71]. Undoubtedly, the activation of Raf paralogs is complicated and largely remains convoluted [72]. Our findings unveil that Rab1A constrains the activation of Raf‐1 depending on its GTPase activity, which sheds light on the mysterious regulation of the Ras‐Raf module. Therefore, the mechanistic exploration of Raf paralogs and Rab1A, as well as their role in *vivo*, needs to be delineated. It should be acknowledged that this study has certain limitations. Due to logistical constraints during the experimental procedure, liver weight and liver‐to‐body weight ratio data were collected from only half of the animals (*n* = 4 per group) in the inducible whole‐body knockout model. Although we performed an independent replication experiment to corroborate these findings, the reduced sample size in the initial cohort remains a practical limitation of the present study.

In conclusion, our study uncovers a previously undescribed population of LAHs that correlates with MASLD/MASH progression. As Rab1A is shown to be the prominent player in LAHs, we further reveal the role of Rab1A in favoring HFD‐induced hepatic steatosis under a pathophysiological context. Our findings provide a novel insight into the pathobiology of MASLD on HFD. In addition, we delineate the role of the Rab1A‐Raf‐1‐ERK1/2‐PINK1 signaling module in mitophagy and MASLD/MASH progression, thereby highlighting this axis as a potential target for future therapeutic development.

## 4 Materials and Methods

### 4.1 Animals, Treatments, and Diets

All the animal care and treatments were carried out in compliance with protocols approved by the Institutional Animal Care and Use Committee (IACUC) of Shanghai Jiao Tong University (IRB No. A2021016). Rab1A^Flox/Flox^ and whole‐body knockout mice were prepared as described in our previous study [28]. Briefly, Rab1A^Flox/+^ mice were self‐crossed to generate homozygous Rab1A^Flox/Flox^ mice, which were then mated with ROSA‐CreERT^2^/ERT^2^ to generate ROSA‐CreERT^2/+^; Rab1A^Flox/+^ mice. ROSA‐CreERT^2/+^; Rab1A^Flox/+^ mice were further backcrossed to generate ROSA‐CreERT^2/+^; Rab1A^Flox/Flox^ mice. Genotyping was performed as described elsewhere. ROSA‐CreERT^2/+^; Rab1A^Flox/Flox^ and the control mice were intraperitoneally injected with tamoxifen for 5 consecutive days at a concentration of 20 mg/mL to generate Rab1A knockout and wild‐type control mice. The knockout efficiency of Rab1A was evaluated by immunoblot and qRT‐PCR using tissues from the sacrificed mice of both groups.

For the Western diet‐fed model, six‐week‐old mice were injected with tamoxifen to induce Rab1A knockout as described above. One week later, the mice were fed ad libitum for 16 weeks on either a standard chow (7% fat control diet, TD.170522, ENVIGO) or a Western diet (45% kcal Fat Diet, TD.08811, ENVIGO). The mice were divided into 4 groups: (1) wild‐type and (2) Rab1A knock‐out group fed with a normal chow diet, (3) wild‐type and (4) Rab1A knock‐out group fed with a Western diet, *n* = 8 for each group. The body weight and fasting blood glucose were monitored weekly as described elsewhere. Due to a procedural coordination issue, liver weight data in this cohort were obtained from 4 mice per group; an independent replication cohort was subsequently analyzed to confirm these findings. Detailed sample sizes and exclusion criteria for all measurements performed in the inducible whole‐body Rab1A knockout mouse model are provided in Table S1A.

For the compound treatment experiments, 7‐week‐old male C57BL/6J mice (purchased from Charles River, Vital River) were used in this study. All the mice were divided into 4 groups: (1) normal chow diet, (2) Western diet + Vehicle, (3) Western diet + Urolithin A (MedChemExpress, HY‐100599), (4) Western diet + C16‐PAF (TargetMol, T21547), *n* = 5 for the normal chow diet and 10 for the other groups. For the Urolithin A treatment, mice were administered intragastrically 5 times/week at 30 mg/kg body weight. For the C16‐PAF treatment, the mice were intraperitoneally injected at 0.5 mg/kg body weight every other day. Vehicle (0.1% Tween80) was used in control mice. Both compounds were administered for 12 consecutive weeks. All the mice in the treatment groups were maintained on a Western diet for at least 16 weeks unless specified.

### 4.2 Cell Culture

AML12 (RRID: CVCL_0140), 293T (RRID: CVCL_0063) and NIH/3T3 (RRID: CVCL_0594) cell lines were purchased from the National Collection of Authenticated Cell Cultures (Academy of Sciences, Shanghai, China). MEF (RRID: CVCL_C1M8) cells were isolated from mouse embryos as described elsewhere [73]. All cell lines used in this study were free from contamination by other cell lines or mycoplasma. AML12 cells were maintained in DMEM/F12 (Gibco, 11320033) supplemented with 10% fetal bovine serum (FBS, Biowest, S1560), 1% ITS (Gibco, 41400‐045) and 40 ng/mL Dexamethasone (Sigma Aldrich, D4902). NIH/3T3, 293T, and MEF cells were maintained in DMEM (Gibco, 10566‐016) medium supplemented with 10% FBS. For the palmitic acid (PA) treated cell model, cells were treated with BSA‐PA to mimic high fat diet treatments in mice as described previously with minor modification [47, 48]. Briefly, AML12 cells were serum‐starved for 6 h with DMEM/F12 media without FBS, then switched to DMEM/F12 complete media plus 50µM BSA‐Palmitate Saturated Fatty Acid Complex (Cayman, 29558) for 48 h. For the insulin stimulation assay, the cells were treated as described elsewhere [74, 75]. AML12 cells were serum‐starved overnight, then switched to DMEM/F12 media supplemented with 3 or 50 nm insulin for 6 h as indicated.

### 4.3 Human Liver Samples

Our research was conducted in accordance with both the Declaration of Helsinki and the Declaration of Istanbul. Human liver tissues were obtained from patients undergoing liver transplant surgery or cholecystectomy at the Huashan Hospital affiliated with Fudan University, Shanghai, China. Prior to the study, all patients provided written informed consent for the collection of research biopsies, and the protocols were approved by the local ethics committee (KY2021‐856 by HIRB of Huashan Hospital, Fudan University). In total, 38 patients participated in the current study. The inclusion criteria were patients aged 18 to 71 years, histologically confirmed MASLD based on liver biopsy, and availability of complete clinical and biochemical data. The exclusion criteria included the presence of other chronic liver diseases (e.g., chronic hepatitis B or C, autoimmune hepatitis, or primary biliary cholangitis), significant alcohol consumption (>30 g/day for men and >20 g/day for women), evidence of drug‐induced liver injury and a history of malignancy or significant systemic comorbidities. The demographic and clinical information about the in‐house cohort was shown in Tables S2 and S3.

### 4.4 Bulk and Single‐Cell/Nucleus RNA Expression Analysis of Public Datasets

The bulk RNA‐seq (GSE130970, GSE225740, and GSE167523), single‐cell RNA‐seq (GSE218299) [32], and single‐nucleus RNA‐seq (GSE202379 [38] and GSE289173[PMID: 40562914]) datasets were obtained from the Gene Expression Omnibus (GEO) website (https://www.ncbi.nlm.nih.gov/geo). The genes of the bulk RNA‐seq data were annotated and analyzed using R v4.3.0. *RAB1A* expression levels were plotted across the histological MASLD severity spectrum (0–3 & 4–7) in R v4.3.0. The difference was tested by the Wilcoxon test. The count matrices of the single‐cell/nucleus RNA‐seq data were used for this analysis. The cells with low quality (UMI (Unique Molecular Identifier) count < 500, UMI count > 5000, or mitochondrial percentage > 10%) were excluded. The cells from different samples were integrated using the R Harmony package and further analyzed by the R Seurat package (Seurat/Harmony pipeline) [76, 77]. Specifically, the gene expression levels were normalized by the LogNormalize method, and the top 3000 most variable features were selected for scaling and principal component analysis (PCA, *n =* 20). The cell embeddings were corrected by the Harmony method, and dimensionality was reduced using Uniform Manifold Approximation and Projection (UMAP) based on the Harmony dimensions. The shared nearest neighbor (SNN) graph was constructed based on the Harmony cell embeddings, and cell clusters were identified by an SNN modularity optimization‐based clustering algorithm. The cell clusters were annotated using the marker genes of the previous study [32]. The hepatocytes were further subclustered using the R Seurat/Harmony analysis pipeline. The LAHs in mouse samples were identified based on their higher percentage in the samples with HFD. The LAHs‐like clusters in human samples were identified based on the enrichment of the DEGs in the LAHs cluster (Fisher's exact test, *P* < 0.05), as well as the high expression of LAHs marker genes (*PGK1*, *ACTB* and *RAB1A*). The cell subset proportions were calculated for diet types or samples, and the difference was tested by the Chi‐square test for mouse data and by the Wilcoxon test for human data. The LAHs and non‐LAHs scores were calculated by averaging the normalized expression levels of the DEGs and were subsequently normalized to Z‐scores. The adipogenesis score was computed based on genes involved in the ‘adipogenesis’ pathway (WikiPathway database) using the AddModuleScore function implemented in the R Seurat (v4.4.0) package. The pathway enrichment analysis was conducted using the R clusterProfiler package, using the pathways and genes curated by MSigDB [78, 79]. All samples from the snRNA‐seq data were strictly reclassified according to *SAF* scoring criteria: samples with *SAF* = 0 were defined as no‐MASLD; cases with *S *≥ 1, *A* < 2 (regardless of F) were defined as MASLD; cases with *S* > 1 and *A* ≥ 2 (regardless of F) were defined as MASH; and livers from patients with liver failure or hepatocellular carcinoma secondary to fatty liver disease were classified as end‐stage. The summary of human cohorts used in this study is provided in Table S2.

### 4.5 AAV‐Mediated Rab1A Knockdown in Liver

To knock down Rab1A specifically in the liver, AAV9‐shRab1A with the liver‐specific promoter TBG (synthesized by Hanbio, Shanghai, China) particles were intravenous injected into 7 weeks old C57BL/6J male mice. Scrambled shRNA was used as a negative control herein. The knockdown efficiency was validated by Western blot and qRT‐PCR. The mice were divided into 2 groups (*n* = 8): AAV9‐Scramble + Western diet and AAV9‐shRab1A + Western diet. The Western diet was maintained for at least 16 weeks for the diet‐induced obesity model. During the establishment of WD‐fed models, one mouse from each of the control and shRab1A groups was excluded due to severe injuries from cage‐mate fighting, yielding an effective sample size of seven mice per group. Detailed sample sizes and exclusion criteria for the remaining measurements in the control and hepatic Rab1A‐knockdown cohorts are listed in Table S1B.

### 4.6 Glucose and Insulin Tolerance Test

Metabolic studies were performed according to the recommendations of the Mouse Metabolic Phenotyping Center (MMPC) Consortium [80]. For the GTT assay, 6 h‐fasted mice (morning fast) were subjected to an intraperitoneal injection of glucose at 2 mg/g body weight. Blood glucose levels were measured using a glucometer (Roche, Accu‐Chek) at the indicated time points (0, 30, 60, 90, and 120 min) after glucose injection. For the ITT assay, mice were fasted for 4 h and then injected intraperitoneally with insulin (Humulin R, Eli Lilly) at 0.75 U/kg body weight. Tail blood glucose levels were measured at indicated times after insulin injection.

### 4.7 Hepatic Biochemical Evaluation

Serum and tissue levels of ALT, AST, cholesterol, and triglycerides were measured using respective colorimetric kits (Elabscience), according to the manufacturer's protocol. The ALT and AST enzyme concentrations were expressed in international units. For in vivo circulating insulin measurement, blood was collected from the tail vein 30 min after intraperitoneal injection of glucose (2 g/kg body weight). Blood samples were centrifuged, and serum was collected for the measurement of insulin concentrations using the Insulin ELISA kit (Mercodia, #10‐1247‐01) following the manufacturer's instructions.

### 4.8 Plasmids, shRNA, Lentivirus Packaging and Stable Cell Lines

Mouse Rab1A tagged ORF (Myc‐Flag‐tagged‐Rab1A) and PINK1 cDNA ORF (PINK1 C‐Myc tag) were purchased from OriGene Technologies (Cat#: MR202153) and Sino Biological (Cat#: MG52375‐CM), respectively. The mouse Raf1 DNA sequence was amplified by PCR from cDNA reverse‐transcribed from AML12 cells, verified by DNA sequencing, and inserted into pCMV‐C‐HA (Beyotime Biotech, Shanghai, China) and pcDNA3.1‐StrepII (Addgene, #208617) plasmids to generate HA‐Raf1 and Strep‐Raf1. Similarly, the mouse ERK1 and ERK2 DNA sequences were constructed into pcDNA3.4‐N‐Flag plasmids using XhoI and BamHI restriction enzyme sites. The validated mouse Rab1A and PINK1 shRNAs were synthesized at Sangon Biotech (Shanghai, China) and cloned into the lentiviral vector pLKO.1‐TRC (Addgene, #10878) using AgeI and EcoRI restriction enzyme sites. Three validated shRNAs against Rab1A were shown here. #506: GGAGTCCTTCAATAACGTTAA; shRab1A #686: AAGAA CGCAACGAATGTAGAA; shRab1A #771: GCCGAGAAGTCCAATGTTAAA. Flag‐Rab1A^S25N^ and Rab1A^Q70L^ mutations were generated from Myc‐Flag‐tagged‐Rab1A using the QuikChange site‐directed Mutagenesis Kit (Agilent Stratagene). Rab1A‐wt (wild type), Rab1A^S25N^, and Rab1A^Q70L^ used for rescue experiments were constructed in lentiviral vector pCDH‐EF1‐coGFP (Systembio) from XbaI and BamHI restriction enzyme sites. The PINK1‐wt (wild type), PINK1^S228A^ and PINK1^S228E^ for reconstitute experiments were established in lentiviral vector pLVX‐Blasticidin (Addgene) backbone. The lentivirus was prepared as described in the previous study. For the Rab1A stable knockdown cells, AML12 cells were transfected with the pLKO.1‐shRab1A lentivirus and selected with puromycin at 1 µg/mL for 2 weeks. For Rab1A rescued stable cells, the Rab1A knocked‐down AML12 cells were transfected lentivirus carrying optimized Rab1A‐wt, Rab1A^S25N^ and Rab1A^Q70L^, and then sorted by FACS for stable cell lines establishments. The PINK1‐C‐Myc truncated mutants were amplified by PCR using respective primers and cloned into pEGFP‐N1‐3Myc plasmids. The primers for cloning were listed in Table S4.

For AAV9‐shRab1A construction, validated shRNA against Rab1A sequences were ligated into the pHBAAV‐TBG‐MCS‐P2A‐zsgreen vector with MluI and HindIII restriction enzyme sites. AAV particles were prepared by co‐transfecting pAAV‐RC (10 µg), pHelper (20 µg), and pHBAAV‐TBG‐MCS‐P2A‐shRab1A (10 µg) together with Lipo3000 in AAV‐293 cells (100 mm dish). The virus was harvested and purified for further use after 72 h.

### 4.9 In Vitro IP‐Kinase Assay

The in *vitro* immunoprecipitation‐kinase assay was performed as previous studies described with minor modifications [81, 82]. In brief, Flag‐Rab1A (*wt*, *S25N* and *Q70L*) and Strep‐Raf1 were transfected into HEK293T cells, respectively, using Lipofectamine 3000 as instructions indicated. Cells were harvested and lysed for immunoprecipitation after 48 h. The cells were suspended in RIPA buffer (25 mm Tris‐HCl pH 7.5–8, 150 mm NaCl, 1 mm EDTA, 1 mm EGTA, 0.5% Triton X‐100, 0.5% NP‐40, 0.5% Sodium deoxycholate, 0.1 mm MgCl_2_) for cell lysis, and then subjected to sonication for thorough lysis. The supernatant of cell lysis was collected for purification of target proteins. Anti‐FLAG M2 magnetic beads (Sigma‐Aldrich) were used for purifying Flag‐Rab1A proteins as the manufacturer's instructions indicated. Strep‐Tactin resin (QIAGEN, 2‐1206‐025) was applied for purifying Strep‐Raf1 as the instructions described. The purity of immunoprecipitated proteins was evaluated by running SDS‐PAGE gels and staining with Coomassie blue R250. Flag‐Rab1 and Strep‐Raf1 were mixed in the 1× kinase buffer (25 mm Tris‐HCl pH 7.5, 10 mm MgCl_2_, 2 mm DTT, 0.1 mm Na_3_VO_4_, 200 µm ATP) at the indicated amounts, and then incubated for 30 min at 30°C. The reaction mixture was analyzed by immunoblots and then followed by an ADP‐Glo kinase assay (Promega, V6930) to quantify the *IC*_50_ value as the manufacturer's instructions indicated.

### 4.10 Proteomic and Phosphoproteomic Analyses

Scramble and shRab1A AML12 cells (2 × 10^7^) were treated with 50 µm PA for 48 h as described above. The cells were washed with ice‐cold 1 × PBS and collected by cell scrapers, and then fast‐frozen by liquid nitrogen after centrifuge. The cells were subjected for 4D label‐free proteomic and phosphoproteomic analyses as previously described [83].

For proteomic analysis, the cells were lysed with SDT buffer (4% SDS, 100 mm Tris‐HCl, 1 mm DTT, pH 7.6) and quantified with the BCA protein assay kit (Bio‐Rad, USA). Protein (20 µg) for each sample was separated on a 4%‐ 20% SDS‐PAGE gel and visualized by Coomassie Blue R‐250 staining. Trypsin was used to digest the protein according to the filter‐aided sample preparation (FASP) procedure as described by Matthias Mann [84]. The digests of each sample were desalted on C18 Cartridges (Empore SPE Cartridges C18 (standard density), bed I.D. 7 mm, volume 3 mL, Sigma), concentrated by vacuum centrifugation, and reconstituted in 40 µL of 0.1% (v/v) formic acid. The peptide content was estimated by UV spectral density at 280 nm using an extinction coefficient of 1.1 of 0.1% (g/L) solution that was calculated on the basis of the frequency of tryptophan and tyrosine in vertebrate proteins. LC–MS/MS analysis was performed on a timsTOF Pro mass spectrometer (Bruker) that was coupled to Nanoelute (Bruker) as described elsewhere.

For phosphoproteomic analysis, the samples were handled using similar procedures as the proteomic analysis in terms of protein extraction and digestion. The phosphopeptides were enriched by the IMAC approach using the High‐Select Fe‐NTA Phosphopeptides Enrichment Kit according to the manufacturer's instructions (Thermo Scientific). After lyophilization, the phosphopeptides were resuspended in 20 µL loading buffer (0.1% formic acid) for LC‐MS analysis using a Proxeon‐nLC 1000 (Thermo Scientific) provided by Shanghai Applied Protein Technology Co. Ltd (Shanghai, China).

All the MS raw data for each sample were combined and searched in the UniProt public database (http://www.uniprot.org/) using the MaxQuant 1.6.14 software for identification and quantitation analysis. The bioinformatic analysis, including KEGG annotation, Enrichment analysis, and Volcano plot, was performed using respective packages in RStudio.

### 4.11 Transmission Electron Microscopy

Scramble and shRab1A cells were seeded in each well of 6‐well plates the evening before sample preparation. The cells were serum‐starved for 6 h and then switched to DMEM/F12 complete media supplemented with 1% ITS, 40 ng/mL Dexamethasone and 50 µm PA. Forty‐eight hours later, the cells were fixed with 2.5% glutaraldehyde in 0.1 m sodium cacodylate buffer pH 7.4 for 20 min at room temperature. The fixed samples were post‐fixed with 2.5% glutaraldehyde for another 1 h at 4°C. The samples were washed with 0.1 m PB three times (3 min/each) and dehydrated in an ethanol series (30%, 50%, 70%, 85%, 95% ethanol in distilled water, each for 10 min). Then the samples were dehydrated in pure ethanol two times (each for 20 min) and infiltrated with ethanol: epoxy 812 (2: 1, 1: 1, 1: 2, each for 45 min). The samples were infiltrated with pure epoxy 812 overnight and then infiltrated with fresh epoxy 812 for another 1 h and embedded in epoxy 812. After polymerizing at 65°C for 48 h, the resin blocks were trimmed and thin sectioned to a thickness of 70 nm on an ultramicrotome (Leica UC7) using a diamond knife. The thin sections were mounted onto formvar‐coated copper grids and counterstained with 3% uranyl acetate in 70% methanol and 30% water for 7 min, followed by lead citrate for 3 min, and viewed at 120 kV in a transmission electron microscope (Thermo Fisher/FEI Talos L 120C). Mitochondrial morphometry was performed on TEM micrographs using ImageJ/Fiji software according to the protocols published elsewhere [85, 86]. Mitochondrial length was determined by calculating the maximum Feret diameter of manually traced outer membranes. For IMM cristae quantification, the number of distinct cristae per mitochondrion was normalized to the mitochondrial area (Cristae Density). All measurements were performed by an investigator blinded to the treatment groups.

### 4.12 Histology, Immunofluorescence Staining and Confocal Microscopy

For Hematoxylin and Eosin (H & E) staining, the liver and pancreas tissues were fixed with 4% formaldehyde, embedded in paraffin, and cut into 6 µm serial sections. H&E staining was performed following the standard procedures as described elsewhere. For the immunohistochemistry staining, the paraffinized sections (6 µm) were stained using Rab1A (Proteintech, 1: 200), p‐ERK1/2(Thr202/Tyr204) (Proteintech, 1: 400), p‐Raf1‐S338 (Abclonal, 1: 300) and p‐PINK1(S228) (Affinity Biosciences, 1: 100). Citrate buffer (10 mm sodium citrate, 0.05% Tween 20, pH 6) was applied to the antigen retrieval procedure in a pressure cooker for 20 min. The IHC staining protocol was followed as described in previous studies. For the immunofluorescence staining, cells were seeded on the coverslips in 24‐well plates. After treatments, the cells were fixed in 4% formaldehyde and then blocked with 5% BSA in PBS. 0.25% Triton X‐100 was used to permeabilize the cell membrane for 10 min on ice. Cells were incubated with primary antibodies overnight at 4°C with gentle shaking. The cells were washed with 1 × PBS with 0.1% Tween 20 for 3 times, 5 min each. Appropriate fluorophore‐conjugated Alexa secondary antibodies (Thermo Fisher) were used for staining and mounted in Antifade mounting medium with DAPI (Vector, H‐1000‐10). Alternatively, FlexAble Labeling Kits (Proteintech, #KFA501 and 502) were used for both Rabbit primary antibody detection per the manufacturer's instructions. Confocal images were acquired using an inverted confocal laser scanning microscope, Zeiss LSM 900 or Leica TCS SP8 STED. The quantification of fluorescence staining images was performed using ImageJ software as described elsewhere.

### 4.13 Histological Analysis and Quantification of Cell Size

For the human liver tissues, paraffinized sections were stained with Hematoxylin & Eosin. MASLD activity score was assessed by 3 certified liver pathologists blinded to the clinical details according to the NAFLD activity score (NAS) system developed by the NASH Clinical Research Network [87, 88]. Briefly, the NAS is the sum of three components: Steatosis (0–3): Quantifies the percentage of hepatocytes containing fat droplets (<5% to >66%); Lobular Inflammation (0–3): Assesses the number of inflammatory foci per 200× field; Hepatocyte Ballooning (0–2): Evaluates the presence and prominence of enlarged, degenerating hepatocytes. A total NAS of ≥5 is diagnostic of steatohepatitis (MASH/NASH), while scores <3 suggest simple steatosis. This standardized approach allows for a rigorous, semi‐quantitative comparison of disease severity across clinical specimens or experimental models. The scoring of immunohistochemistry staining of Rab1, p‐Raf1 (S338), and p‐ERK1/2(Thr202/Tyr204) was evaluated by 3 pathologists blinded to the clinical information as well. The H score ranges from 0 to 300 depending on the IHC staining intensity and pattern.

For the Oil Red O staining, the mouse liver tissues were embedded in OCT (Sakura) and cut into frozen sections at 10 µm. The slides were fixed in formalin and washed with tap water for 5 min. After rinsing with 60% isopropanol, the sections were stained with freshly prepared Oil Red O working solution for 15 min. The nuclei were stained with hematoxylin for 3 min and washed with tap water till colorless. The sections were mounted with an appropriate medium for analysis.

### 4.14 BODIPY, Nile Red, and Mitophagy Detection

For BODIPY staining, the cells were seeded in a 35 mm dish with a glass bottom (0.17 mm) and treated with 50 µm PA as mentioned above. At the time point of interest (typically 48 h), 2 µm BODIPY 493/503 dye (Thermo Fisher) was added to the medium. After 30 min incubation, the cells were fixed with 4% formaldehyde for 15 min at room temperature. Then the cells were observed under a fluorescence Microscope. Nile red (MCE, Shanghai, China) staining was performed similarly to the above description.

Mitophagy was evaluated with a Mitophagy detection kit according to the manufacturer's instructions (Dojindo, Cat: #MD01). Briefly, cells were seeded for the assay. The culture media was removed, and the cells were washed with serum‐free DMEM/F12 media twice. Mitophagy Dye working solution (100 nm) was added and then incubated at 37°C for 30 mins. The supernatant was discarded and replaced with DMEM/F12 complete medium with 50 µm PA‐BSA or U0126 at 37°C for 36 h. After removing the supernatant, Lysosome Dye (1 µm) and Mitotracker Deep Red (100 nm, MCE, Shanghai, China) were added to the cells and incubated for 30 mins. The cells were washed with serum‐free DMEM/F12 medium twice and then observed by confocal fluorescence microscopy.

For mitochondrial staining, Mitotracker (Red, 579 nm, Thermo Fisher) was diluted in Opti‐MEM (Reduced serum, Gibco, 1: 2000) and incubated for 30 min at 37°C. The cells were washed with PBS twice with gentle shaking and then fixed with 4% formaldehyde for 15 min at room temperature.

### 4.15 Proximity Ligation Assay

Duolink Proximity Ligation Assay (PLA) was performed as described in a previous study. AML12 cells were used for endogenous Rab1A & Raf1 and ERK1/2 & PINK1 interactions as the manufacturer's instructions indicated. NIH/3T3 cells were used for exogenous Flag‐Rab1A and HA‐Raf1 interactions. Flag‐ERK1/ERK2 and PINK1‐C‐Myc plasmids were co‐transfected into MEF cells for exogenous interactions by PLA. In brief, the cells were seeded on glass slides and fixed at appropriate confluency with 4% formaldehyde. Cells were permeabilized with 0.25% Triton X‐100 in PBS for 10 min on ice, and then blocked using Blocking Solution for 60 min at 37°C. Primary antibodies (anti‐Rab1A, rabbit from Proteintech, 1: 250; anti‐Raf1, mouse from Abmart, 1: 300; anti‐Flag, rabbit from Proteintech, 1: 300; anti‐HA, mouse from cell signaling technology, 1: 250; anti‐ERK1/2, mouse from Proteintech, 1: 200; anti‐PINK1, rabbit from Proteintech, 1: 300; anti‐Myc, mouse from cell signaling technology, 1: 200) were diluted with antibody diluent, and added to each sample. The samples were incubated in a humidified chamber for 60 min at 37°C. Duolink PLA PLUS and MINUS probes (DUO92005, DUO92001, Sigma‐Aldrich, USA) were prepared and ligated with primary antibodies after washing in 1× Wash Buffer A. The Duolink in situ detection reagent (green; DUO92014, Sigma‐Aldrich, USA) was employed for amplification. The images were acquired through a fluorescence confocal microscope after a final wash.

### 4.16 Mitochondrial Oxygen Consumption/OCR

Mitochondrial OCR was performed with an Agilent Seahorse XF analyzer system combined with the Seahorse cell mito stress test kit (Agilent, Santa Clara, USA) according to the user guide. A total of 10 000 scramble and shRab1A cells were seeded per well of the Seahorse XFe96 96‐well assay microplate on the evening before the experiments were conducted. The complete culture medium was changed to Seahorse XF DMEM supplemented with 5 mm glucose, 1 mm pyruvate, and 10 mm glutamine 1 h prior to measurement. After incubation in a 37°C incubator for 1 h, oxygen consumption rates were detected under basal, oligomycin (1.5 µm), FCCP (1 µm), and Rotenone/Antimycin A (0.5 µm) conditions. OCRs were normalized to total cell number determined by DAPI staining, which was quantified at the end of the experiments.

### 4.17 Extraction of Mitochondria

Mitochondria were isolated using the mitochondrial extraction kit per the manufacturer's instructions (Solarbio Life Science, China). Scramble and shRab1A cells were seeded in 15 cm dishes and harvested with 0.25% trypsin when the cell confluency reached 95%. The cells were centrifuged and washed with PBS twice. Mitochondria were extracted following the instructions at different centrifugal speeds. Mitochondrial protein was prepared by sonication of the extracted mitochondria in RIPA buffer (25 mm Tris‐HCl pH 7.5–8, 150 mm NaCl, 1 mm EDTA, 1 mm EGTA, 0.5% Triton X‐100, 0.5% NP‐40, 0.5% Sodium deoxycholate, 0.1 mm MgCl_2_).

### 4.18 Structural Prediction by AlphaFold

The structural interaction between Rab1A and Raf‐1 was predicted using AlphaFold v2.3.1 as described elsewhere [89, 90]. Briefly, sequences of the two proteins were prepared by querying the UniProt database. The multimer model was then selected for the run, since the prediction was for a complex structure. An NVIDIA A100 graphics card (80GB video memory) was configured in support of efficient operation of AlphaFold for complex prediction. The number of models was set to 1. ChimeraX was next used to analyze the prediction results of AlphaFold. pLDDT (predicted local distance difference test) and PAE (predicted Aligned Error) were two important parameters for interpreting the prediction results. pLDDT is a metric for evaluating model quality, ranging from 0 to 100. In ChimeraX, the pLDDT value of each residue can be viewed by color coding. PAE is a metric for evaluating model accuracy, which indicates the difference between the predicted distance between two residues and the actual distance. The smaller the PAE value, the closer the predicted distance is to the true value. The quality and accuracy of protein structures predicted by AlphaFold were assessed by analyzing pLDDT and PAE.

Three‐dimensional structures of ERK1/2 and PINK1 were predicted using the AlphaFold3 server (https://alphafoldserver.com) [91]. Amino acid sequences of ERK1/2 and PINK1 were submitted to the server using default parameters. Predicted protein structures were visualized and analyzed using PyMOL software (Version 2.5.0, Schrödinger LLC). Interaction interface parameters and binding free energy (Δ^i^G) of the ERK1/2‐PINK1 complex were calculated using PDBePISA (https://www.ebi.ac.uk/pdbe/pisa/) [92].

### 4.19 Coimmunoprecipitation and Immunoblot

For the FLAG‐beads IP/co‐IP assay, Flag‐Rab1A, ERK1/2 and/or HA‐Raf1, PINK1‐C‐Myc plasmids (1:1) were transfected into 293T cells for 40 h with Lipofectamine 3000 according to the manufacturer's instructions. Cells were harvested and washed with ice‐cold PBS twice, and then lysed with the aforementioned RIPA buffer with protease inhibitor cocktail (EDTA‐free, Roche) for 30 min on ice, with vortexing for 15 s every 10 min followed by sonication. Supernatants were collected after centrifugation. Cell lysates were incubated with Anti‐FLAG M2 Magnetic beads (Sigma‐Aldrich) overnight at 4°C. The next day, beads were washed with RIPA buffer with 0.5% Tween 20 three times, 10 min each at 4°C. The complexes were eluted with 1X loading buffer by heating for 10 min at 70°C for further Western blot analysis. For the endogenous IP assay, primary Rab1A and ERK1/2 antibodies were incubated with Dynabeads Protein G (Thermo Fisher) for 30 min prior to use. The other procedures were performed likewise.

Detailed information on the antibodies used for Western blot, immunofluorescence, and immunohistochemistry is provided in Table S5. Primary antibodies were used according to manufacturers’ instructions overnight at 4°C unless otherwise indicated. Secondary antibodies were used at a dilution of 1: 5000 for 1 h at room temperature. For immunoblotting, cell and tissue protein extracts were separated by SDS‐polyacrylamide gel electrophoresis (SDS‐PAGE) and were transferred onto PVDF membranes (Bio‐Rad). Membranes were blocked with 5% skim milk for 1 h at room temperature and then incubated with primary antibodies overnight at 4°C. Antibodies were diluted according to the manufacturer's instructions. Membranes were further incubated with HRP‐conjugated secondary antibodies, followed by enhanced chemiluminescence detection reagents (NEL105001EA, Perkin Elmer) and analyzed using the ChemiDoc XRS+ system (Bio‐Rad). Data were quantified using the Image Laboratory v.6.0.1 Software. The quantification of immunoblot bands was performed using ImageJ software as described elsewhere.

### 4.20 Reverse Transcription and Quantitative PCR

Total RNA was extracted from cells or tissues using TRIzol reagent (Thermo Fisher) according to the manufacturer's instructions. cDNA synthesis was conducted using the GoScript reverse transcription system (Promega) per the manufacturer's instructions. Quantitative PCR was performed with SYBR Green PCR master mix (Applied Biosystems) using a QuantStudio 7 Flex Real‐Time PCR System (Applied Biosystems). β‐actin was used as the internal control. Data were normalized using the average *Ct‐*value of the internal control and further analyzed by the 2^−ΔΔ^ *^Ct^* method.

To determine the mtDNA content, DNA was extracted from the whole‐cell genome or isolated mitochondria using the TIANquick Midi Purification Kit (TIANGEN) according to the manufacturer's instructions. mtDNA amounts were quantified by qPCR using Taq Pro Universal SYBR qPCR Master Mix (Vazyme) and a Thermo Fisher QuantStudio 3. Measurements were normalized to 18S rRNA. The primers for qPCR were listed in Table S4.

### 4.21 Statistical Analysis

Data were evaluated for potential outliers using the Grubbs’ test (extreme studentized deviate method) with an alpha level of 0.05. Outliers identified by this method were excluded from the final analysis. Where necessary, data were normalized to the respective control group to account for inter‐assay variability. No other data transformations were applied. All data were presented as mean ± SEM (or mean ± SD where indicated), as specified in the figure legends. The sample size (*n*) for each experiment represents biological replicates and is specified in the corresponding figure legends. The validity of statistical assumptions was verified prior to testing: normality was assessed using the Shapiro–Wilk test, and homogeneity of variance was evaluated using Levene's test (*P *> 0.05 in both cases confirmed that the assumptions of normality and equal variance were met, justifying the use of the parametric tests below). For comparisons between two groups, a two‐tailed unpaired Student's *t*‐test was employed. For experiments involving multiple groups, one‐way ANOVA followed by Tukey's post‐hoc test was used for multiple comparison adjustments. For time‐course metabolic data (e.g., GTT and ITT), two‐way ANOVA with Sidak's post‐hoc test was utilized to assess differences between genotypes or treatments over time. The area under the curve (*AUC* values) of ITT and GTT in the figures was determined by the trapezoidal rule and compared using a two‐tailed unpaired Student's *t*‐test (two groups) or one‐way ANOVA with Tukey's post‐hoc test (multiple groups). The testing level was set at alpha = 0.05, and a two‐sided *P*‐value of <0.05 was considered statistically significant across all analyses. Exact *p*‐values and specific statistical tests used for each panel are detailed in the figure legends. Statistical analyses were performed using GraphPad Prism (v.7.0) and Microsoft Excel (v.2016).

## Author Contributions

Xin Zhang conceptualized and directed the overall project. Xin Zhang, Li Zhang, Jianhua Li, and Huilu Zhang designed and/or performed experiments and analyzed data. Li Zhang performed the bioinformatic analyses. Jianhua Li, Chao Sun, Huilu Zhang, Qi Qin, and Binbin Li performed mouse and human liver tissue pathological analyses. Zhengxin Wang and Jianhua Li provided the human liver tissues. Xin Zhang, Yichen Huang, Huilu Zhang, and Xiaofan Tian performed biochemical and in vitro experiments in cells. Xin Zhang, Li Zhang, and Jianhua Li prepared figures. Xin Zhang and Zhengxin Wang provided funding and research space. Xin Zhang and Li Zhang wrote the manuscript. Jianhua Li, Huilu Zhang, Xiaofan Tian, and Binbin Li edited the manuscript.

## Funding

This work was supported by the grants from the National Key Research and Development Program of China (2023YFC2505900 to Z.W.), National Natural Science Foundation of China (No. 32100950 to X.Z. and 81873874, 82071797 and 82241225 to Z.W.), and grants from Data Sharing and Emulation of Clinical Trials, CCS‐DASET (SHDC2024CRI069 to J.L.).

## Conflicts of Interest

The authors declare no conflicts of interest.

## Supporting information




**Supporting File 1**: advs77809‐sup‐0001‐SuppMat.docx.

**Supporting File 2**: advs77809‐sup‐0002‐SuppMat.zip.

## Data Availability

The proteomics datasets generated with PA‐treated AML12 cells are available upon request. R scripts and code for the analysis of scRNA‐seq/snRNA‐seq, bulk RNA‐seq, proteomic, and phosphoproteomic datasets are available upon request.
