# Supplementary material for: Rab1A Promotes Hepatic Steatosis by Suppressing Mitophagy via the Raf‐1/ERK1/2/PINK1 Signaling Axis
Source: Adv Sci (Weinh). 2026 Sep 27:e17520. Online ahead of print. doi: 10.1002/advs.202517520 (PMC13616366; doi:10.1002/advs.202517520)
Supplement: Supplementary file 1 [file ADVS-9999-e17520-s001.docx]

**Supplemental Information**

**Fig. S1.**

**Fig. S1, relating to Fig. 1. scRNA-seq analyses uncover increased Rab1A expression in lipid-associated hepatocytes.**

(A) Bubble plot of the expression of cell-type markers for cell annotation.

(B) GSEA showing the upregulated pathways of non-LAHs.

(C) Volcano plots showing the upregulated markers of LAHs. Genes from the upregulated pathways were cross-referenced with 3 bulk RNA-seq datasets from MASLD patients (GSE22740: n = 93, GSE167523: n = 98, GSE130970: n = 78).

(D) Dot plot showing the expression of canonical liver zonation marker genes across the four hepatocyte subpopulations. Rows correspond to hepatocyte subpopulations, and columns to marker genes (GLUL, CYP1A2, CYP2E1, CYP2C8, CYP8B1, CYP2C9, ASS1, ASL, HAL). The color gradient indicates average expression level, and dot size represents the percentage of cells expressing the gene.

(E) The consistency between LAHs-like hepatocytes in human and LAHs in mice, and between non-LAHs in humans and non-LAHs in mice, was inspected by scoring, shown in violin plots. LAHs and non-LAHs scores were calculated by averaging the normalized expression levels of DEGs and then normalizing to a Z-score (Normal: n = 25, MASLD: n = 13, MASH: n = 50, end stage: n = 5). Analyzed by the Wilcoxon test, the *p* value is indicated.

(F) Violin plot showing the Rab1A expression in LAHs-like hepatocytes versus non-LAHs (Normal: n = 25, MASLD: n = 13, MASH: n = 50, end stage: n = 5). Analyzed by the Wilcoxon test, *p* value is indicated.

(G and H) Expression levels of representative adipogenesis-related genes in LAHs/LAHs-like and non-LAHs subpopulations. Left and right panels show mouse and human data, respectively. Dot size indicates the percentage of cells expressing the gene, and color represents the average expression level.

(I and J) The module scores for adipogenesis in LAHs/LAHs-like and non-LAHs subpopulations. Left and right panels show mouse and human data, respectively. Boxplots display the median, boxes represent the 25th and 75th quartiles, and whiskers extend to the minimum and maximum values (Normal: n = 25, MASLD: n = 13, MASH: n = 50, end stage: n = 5). Differences were assessed using the Wilcoxon rank-sum test.

(K) qPCR analysis of Rab1A mRNA levels in healthy controls (n=6) and MASLD patients (n=6). **p*<0.05, unpaired Student’s t test for two groups.

(L) Western blot analysis of Rab1A protein levels in liver tissue lysates from healthy controls (n=6) and MASLD patients (n=6).

(M) Evaluation of the LAHs population by co-staining of Rab1A and PGK1 in liver tissues from healthy controls and MASLD patients (n=3). Representative images are shown. Scale bar, 100 μm, 50 μm and 25 μm.

**Fig. S2.**

**Fig. S2, relating to Fig. 2. Rab1A deficiency alleviates western diet-induced hepatic steatosis in adult mice.**

(A) Validation of Rab1A knockout efficiency after the administration of tamoxifen by IHC staining. Representative images were shown. Scale bar, 50 μm for 4ⅹ and 100 μm for 40ⅹ.

(B) Weekly body weight of WT and Rab1A KO mice on diet. n=8, ****p<0.0001, analyzed by two-way ANOVA.

(C) Representative images of mice in WT and Rab1A KO mice on diet *in situ*. Both male and female groups are shown.

(D) Weekly fasting blood glucose of WT and Rab1A KO mice on diet. n=8, **p<0.01, ***p<0.001, ****p<0.0001, analyzed by two-way ANOVA.

(E) The area under the curve (AUC) of ITT in Figure 2B was determined by the trapezoidal rule. All results are presented as mean ± SEM (n=8). Results were analyzed by unpaired Student’s t test for two groups.

(F) GTT of mice after 16 weeks on diet. All results are presented as mean ± SEM (n=8), *p<0.05, **p<0.01, analyzed by two-way ANOVA.

(G) The area under the curve (AUC values) of GTT in Figure S2F was determined by the trapezoidal rule. All results are presented as means ± SEM (n=8). **p*<0.05, unpaired Student’s t test for two groups.

(H) Representative images of H&E staining of pancreas sections in WT and Rab1A KO mice on diet. Scale bar, 200 μm.

(I) Quantification of circulating insulin of 4 groups of mice on diet. All results are presented as means ± SEM (for ND-WT, ND-Rab1A KO, WD-WT, n=5; WD-Rab1A KO, n=6). **p*<0.05, unpaired Student’s t test for two groups.

(J) Staining of lipid accumulation in liver sections from 4 groups of mice by BODIPY-488. Representative images shown here. Frozen sections were used. Scale bars, 100 μm and 10 μm respectively.

(K) The liver weight of 4 groups of mice on diet. All results are presented as mean ± SEM (n=4). ***p*<0.01, unpaired Student’s t test for two groups.

(L) The liver-to-body weight (B.W.) ratio of 4 groups of mice was calculated here. All results are presented as mean ± SEM (n=4). Results were analyzed by unpaired Student’s t test for two groups.

(M and N) Quantification of triglyceride and cholesterol levels in liver supernatants from WT and Rab1A KO mice on diet. Results are presented as mean ± SEM (n=8). **p*<0.05, unpaired Student’s t test for two groups.

(O) AST from liver tissues of 4 groups of mice was quantified using respective kits. Results are presented as mean ± SEM (n=8). **p*<0.05, unpaired Student’s t test for two groups.

(P) Pathological analysis performed with liver sections from WD-induced control and Rab1A KO mice. Severity of hepatic steatosis is indicated by MASLD Activity Score (NAS). Results are presented as mean ± SEM (n=8). **p<0.01, unpaired Student’s t test.

(Q) Detection of Rab1A knockdown in other organs upon *AAV-shRab1A* delivery by immunoblot.

(R and S) The area under the curve (AUC) of GTT in Fig. 2J and ITT in Fig. 2K was determined by the trapezoidal rule. All results are presented as mean ± SEM (n=7). **p<0.01, ***p<0.001, unpaired Student’s t test.

(T) The liver weight of AAV-Scramble and AAV-shRab1A mice was calculated here. All results are presented as mean ± SEM (n=7) and analyzed by unpaired Student’s t test for two groups. **p*<0.05, unpaired Student’s t test for two groups.

(U and V) Triglyceride (n=6). and ALT levels (AAV-Scramble, n=7; AAV-shRab1A, n=6) in sera of Scramble and Rab1A knockdown mice were quantified using respective kits. **p*<0.05, unpaired Student’s t test for two groups.

(W) Pathological analysis performed with liver sections from WD-induced c Scramble and Rab1A knockdown mice. Severity of hepatic steatosis is indicated by MASLD Activity Score (NAS, n=7). **p<0.01, unpaired Student’s t test.

**Fig. S3.**

**Fig. S3, relating to Fig. 3. Knockdown of Rab1A induces excessive mitophagy and reduced lipid accumulation.**

(A) Images of *shControl* and *shRab1A* AML12 cells under the microscope. White arrows indicate increased vacuoles upon Rab1A knockdown. Scale bars, 200 μm and 50 μm.

(B) Detection of the Golgi apparatus in *shControl* and *shRab1A* AML12 cells by staining with GolgA2 antibody. The fluorescence intensity was quantified using ImageJ. Results are presented as mean ± SEM. Unpaired Student’s t test for two groups. Scale bar, 10 μm.

(C) Immunoblot analysis of ER stress markers (ATF4, CHOP, GRP78/BiP and XBP-1) in *shControl* and *shRab1A* AML12 cells.

(D and E) Quantification of fluorescence intensity in Fig. 3A-B using ImageJ. Results are presented as mean ± SEM. *p<0.05, *** p<0.001, **** p<0.0001, unpaired Student’s t test for two groups.

(F) qRT-PCR analysis of a panel of lipogenesis related genes in *shControl* and *shRab1A* AML12 cells. Repeated independently 3 times, each time in triplicate. Results are presented as mean ± SEM.

(G) Immunoblot analysis of proteins involved in lipogenesis and lipolysis in *shControl* and *shRab1A* AML12 cells.

(H) Representative high magnification electron micrographs of *shControl* and *shRab1A* AML12 cells. Enlarged views of the mitochondria are shown. Scale bars, 5 μm and 2 μm.

(I) Quantification of mitochondrial number per cell for *shControl* and *shRab1A* cells. Results are presented as mean ± SEM. **p*<0.05, unpaired Student’s t test for two groups.

(J) Evaluation of mitochondrial mass by comparing mtDNA to nuclear DNA (nucDNA) through qPCR in *shControl* and *shRab1A* cells. Results are presented as mean ± SEM. **p*<0.05, unpaired Student’s t test for two groups.

(K) Immunoblot analyses of proteins involved in mitochondrial fusion and fission in *shControl* and *shRab1A* AML12 cells.

(L) Evaluation of mitochondrial number and autophagy by immunostaining of MitoTracker and LC3B in *shControl* and *shRab1A* AML12 cells. Fluorescence intensity was quantified using ImageJ. Results are presented as mean ± SEM. ** p<0.01, *** p<0.001, unpaired Student’s t test for two groups. Scale bar, 10 μm.

(M) Assessment of lipid accumulation and mitophagy by immunostaining of BODIPY-488 and LC3B in *shControl* and *shRab1A* AML12 cells treated with 50 μM PA-BSA for 48 hours. Fluorescence intensity was quantified using ImageJ. Results are presented as mean ± SEM. * p<0.05, ** p<0.01, *** p<0.001, unpaired Student’s t test for two groups. Scale bar, 10 μm.

(N) Immunoblot analysis of cytoplasmic and mitochondrial fractionations in shControl and shRab1A AML12 cells. The LC3-II/LC3-I ratio was quantified by ImageJ. Results are presented as mean ± SEM. Unpaired Student’s t test analysis performed.

(O) Acetyl-CoA levels were determined in *shControl* and *shRab1A* knockdown cells after treatment with 50 μM PA-BSA/DMEM at 24, 48 and 72 hours. *p<0.05, unpaired Student’s t test for two groups.

(P) Fatty acid oxidation (FAO) activities were determined in *shControl* and *shRab1A* knockdown cells after treatment with 50 μM PA-BSA/DMEM at 24, 48 and 72 hours. **** p<0.0001, unpaired Student’s t test for two groups.

(Q) ROS levels were assessed by the DCFH-DA probe in *shControl* and *shRab1A* cells after treatment with 50 μM PA-BSA/DMEM at 24, 48 and 72 hours. *p<0.05, unpaired Student’s t test for two groups.

**Fig. S4**

**Fig. S4, relating to Fig. 4. Enhanced Raf/MEK/ERK signaling axis accounts for triggering mitophagy and decreased lipid accumulation upon Rab1A knockdown.**

(A) Immunoblot analysis of activation of the mTORC1/2 and AMPK signaling pathways in *shControl* and *shRab1A* cells.

(B) Differentially expressed proteins between the two groups as shown by a volcano plot. The X-axis indicates the fold change (logarithmic conversion based on 2), and the Y-axis indicates the P value (logarithmic conversion based on 10). Red dots represent up-regulated proteins with significance, while blue dots indicate down-regulated proteins with significance, and gray dots show the proteins without significance.

(C) Immunoblot analysis of components of the RAF/MAPK signaling pathway and phosphorylated PINK1 in *shControl* and *shRab1A* cells combined with or without PA-BSA treatment.

(D) Evaluation of lipid accumulation upon treatments with U0126 by immunostaining in *shControl* and *shRab1A* cells on PA-BSA. Fluorescence intensity was quantified using ImageJ. Results are presented as mean ± SEM. * p<0.05, ** p<0.01, *** p<0.001, **** p<0.0001, unpaired Student’s t test for two groups. Scale bar, 10 μm.

(E) The effect of U0126 on the MEK/ERK signaling pathway was determined by immunoblot of phosphor-ERK1/2.

(F) Evaluation of lipid accumulation upon treatments with the mitophagy inhibitor Mdivi-1 by immunostaining of BODIPY-488 and LC3B in *shControl* and *shRab1A* cells on PA-BSA. Fluorescence intensity was quantified using ImageJ. Results are presented as mean ± SEM. * p<0.05, *** p<0.001, unpaired Student’s t test for two groups. Scale bar, 10 μm.

**Fig. S5**

**Fig. S5,** **relating to Fig. 5. Rab1A suppresses the activation of Raf-1 in a GTPase-dependent manner.**

(A) IP-purified Strep-Raf-1 and Flag-Rab1A stained with Coomassie blue. Asterisks indicate the target bands.

(B) Representative confocal images show the colocalization of Raf-1, Rab1A and its mutants (S25N and Q70L) exogenously in NIH3T3 cells. White arrows indicate the cytoplasmic or nuclear localization upon co-transfection with Rab1A and its mutants. Fluorescence intensity was quantified by ImageJ to show the colocalization of the two proteins. Scale bars, 25 μm.

(C) Immunoprecipitations (IP) of Raf-1 with Rab1A and its mutants were performed in MEF cells. Flag beads were used for IP, p-Raf-1 (S338) and input were detected by respective antibodies.

(D) Immunoblot analysis with the protein samples from *shControl*, *shRab1A* and restored cells by Rab1A WT, S25N and Q70L. The five cell lines were treated with 50 μM PA-BSA for 48 hours.

(E) Evaluation of rescue effects of Rab1A WT and its mutants in Rab1A knockdown cells by staining with Nile Red dye. Scale bar, 20 μm.

**Fig. S6**

**Fig. S6, relating to Fig. 6. ERK1/2 directly regulates PINK1’s phosphorylation via interaction.**

(A) Co-localization of exogenous Flag-ERK1/2 and Myc-PINK1 was evaluated by IF staining and confocal microscopy. Representative confocal images are shown. Fluorescence intensity quantified by ImageJ to show the colocalization of the two proteins. Scale bar, 10 μm.

(B) Interaction between endogenous ERK1/2 and PINK1 was determined by Proximity Ligation Assay (PLA). Green fluorescent dots indicate the closeness of ERK1/2 and PINK1. Scale bar, 10 μm.

(C and D) The confidence and accuracy of the predicted interactions between ERK1/2 and PINK1 were assessed by PAE (predicted Aligned Error). ipTM and pTM values are both used to assess the accuracy of the prediction.

(E) Establishment of stable PINK1-knockdown and PINK1 WT /S228A/S228E rescued AML 12 cells.

(F) Detection of phosphorylation of PINK1 at Ser228 in Urolithin A (10 μM)-treated *shControl*, PINK1-kncokdown and PINK1 WT /S228A/S228E rescued cells.

(G) Immunoblots performed with U0126 treated PINK1^WT^ and PINK1^S228A^ rescued cells. The cells were treated for 24 hours at the indicated concentrations. Phosphorylated ERK1/2 and p-PINK1 (S228) were detected and p-PINK1 (S228) levels were quantified using ImageJ as shown on the right. Results are presented as mean ± SEM. *p<0.05, unpaired Student’s t test for two groups.

(H) Evaluation of mitophagy upon the treatments with Urolithin A (for 48 h) and PA-BSA by immunostaining in PINK1^WT^ and PINK1^S228A^ rescued cells. White speckles indicate occurrences of mitophagy. Scale bar, 20 μm.

**Fig. S7**

**Fig. S7, relating to Fig. 7. Boosting mitophagy via ERK1/2/PINK1 signaling axis lessens lipid accumulation and ameliorates WD-induced hepatic function.**

(A) Chemical structures of Urolithin A and C16-PAF.

(B) Assessment of activation of other MAPKs (p38 MAPK and JNK1/2) upon C16-PAF treatment by immunoblot.

(C and D) Evaluation, by immunostaining, of lipid accumulation and mitochondria affected by Urolithin A and C16-PAF in *shControl* cells. sh*Control* AML12 cells were treated with the indicated concentrations of compounds for 24 hours combined with PA-BSA. BODIPY-488 and MitoTracker label lipids and mitochondria respectively. Scale bar, 20 μm.

(E) Evaluation of lipid accumulation and mitochondria affected by Urolithin A and C16-PAF at the indicated concentrations using immunostaining in *shRab1A* cells. Scale bar, 20 μm.

(F and G) The area under the curve (AUC) of GTT (Fig. 7F) in F and ITT (Fig. 7G) in G was determined by the trapezoidal rule. All results are presented as mean ± SEM (for WD+Vehicle, WD+Urolithin A, WD+C16-PAF, n=10; for Normal chow n=5). **p*<0.05, ***p*<0.01, unpaired Student’s t test for two groups.

(H) Quantification of total cholesterol (TC) levels in sera of 4 groups of mice. Results are presented as mean ± SEM (n=9, 8, 7 and 5 for WD+Vehicle, WD+Urolithin A, WD+C16-PAF, and Normal chow, respectively). **p*<0.05, ***p*<0.01, unpaired Student’s t test for two groups.

(I) Quantification of triglyceride levels in liver supernatants from 4 groups of mice. Results are presented as mean ± SEM (n=6 for WD+Vehicle, WD+Urolithin A, WD+C16-PAF and n=5 for Normal chow). *p<0.05, ** p<0.01, unpaired Student’s t test for two groups.

(J and K) ALT and AST in liver supernatants from the four mouse groups. Data are mean ± SEM (n = 8, 6, 6, and 5 for ALT; n = 8, 7, 8, and 5 for AST; WD+Vehicle, WD+Urolithin A, WD+C16-PAF, and Normal chow, respectively). **p<0.01, ***p<0.001, ****p<0.0001, unpaired Student's t-test.

(L) Evaluation of hepatic steatosis with liver tissues from 4 groups. The dissected livers *in situ* (left panel) and an overview of H&E staining analysis (right panel). Scale bar, 500 μm.

**Fig. S8**

**Fig. S8, relating to Fig. 8. Elevated Rab1A correlates with attenuated activities of Raf-1/ERK1/2/PINK1 in patients with MASLD.**

(A) Evaluation of mitophagy by co-staining of TOM20 and LC3B in high, medium and low NAS score liver tissues. White arrows indicate lipid droplets, yellow arrows indicate mitophagy. Scale bars, Scale bars, 100 μm and 50 μm.

(B) H&E staining of liver tissues from representative high, medium and low NAS score patients. The liver specimens used herein are derived from the same batch of samples as those shown in Fig. 8D. Scale bar, 100 μm.

(C) Correlation plot showing Rab1A and p-ERK1/2(T202/Y204) IHC staining (arbitrary units). Analyzed by a Pearson correlation test, the coefficient of correlation (*r* value), and the *p* value (*P*) are indicated, R squared: 0.7043.

(D) Correlation plot showing p-Raf-1 (S338) and p-ERK1/2 (T202/Y204) IHC staining (arbitrary units). Analyzed by a Pearson correlation test, the coefficient of correlation (*r* value), and the *p* value (*P*) are indicated. R squared: 0.7582.

**Table S1A. Sample sizes and exclusion criteria for control and Rab1A whole-body knockout groups**

**Table S1B. Sample sizes and exclusion criteria for control and shRab1A groups**

**Table S2. Summary of human cohorts used in this study.**

**Table S3. Demographic and clinical characteristics of in-house human participants (Cohort 7)**

**Table S4. Sequences of primers**

| Name Sequences Usage | | |
| --- | --- | --- |
| Primer for Rab1A S25N | forward primer (5’-3’), GGGGTTGGAAAGAACTGCCTTC  reverse primer (5’-3’), GAAGGCAGTTCTTTCCAACCCC | PCR for cloning |
| Primer for Rab1A Q70L | forward primer,  GACACAGCAGGCCTGGAAAGATTTC  reverse primer,  GAAATCTTTCCAGGCCTGCTGTGTC | PCR for cloning |
| Mouse shRab1A #506: | GGTTGGAAAGTCCTGCCTTCT | shRNA construction |
| Mouse shRab1A #686: | GCACAATTGGTGTGGATTTCA | shRNA construction |
| Mouse shRab1A #771: | GGAGTCCTTCAATAACGTTAA | shRNA construction |
| Mouse shPINK1-1 | GCTGCAAATGTGCTGCACTTA | shRNA construction |
| Mouse shPINK1-2 | CCTGGCTGACTATCCTGATAT | shRNA construction |
| Primer for PINK1 S228A | forward primer (5’-3’), CAGGTTCCGCCAGCGAAGCCATCTTA  reverse primer,  GCTGGCGGAACCTGCCGAGATATTCCAC | PCR for cloning |
| Primer for PINK1 S228E | forward primer (5’-3’), CAGGTTCCGAAAGCGAAGCCATCTTA  reverse primer,  GCTTTCGGAACCTGCCGAGATATTCCACATC | PCR for cloning |
| Acly | forward primer,  ACCCTTTCACTGGGGATCACA  reverse primer,  GACAGGGATCAGGATTTCCTTG | qPCR |
| Rab1A | forward primer, CAGTGCTAAGAACGCAACGA  reverse primer,  CTTGACTGGAGTGCTCTGGA | qPCR |
| ACACA | forward primer, ATGGGCGGAATGGTCTCTTTC  reverse primer,  TGGGGACCTTGTCTTCATCAT | qPCR |
| Fasn | forward primer, GGAGGTGGTGATAGCCGGTAT  reverse primer,  TGGGTAATCCATAGAGCCCAG | qPCR |
| Scd1 | forward primer, TTCTTGCGATACACTCTGGTGC  reverse primer,  CGGGATTGAATGTTCTTGTCGT | qPCR |
| Srebf1 | forward primer, GCAGCCACCATCTAGCCTG  reverse primer,  CAGCAGTGAGTCTGCCTTGAT | qPCR |
| Srebf2 | forward primer, GCAGCAACGGGACCATTCT  reverse primer,  CCCCATGACTAAGTCCTTCAACT | qPCR |
| Agpat6 | forward primer, AGCTTGATTGTCAACCTCCTG  reverse primer,  CCGTTGGTGTAGGGCTTGT | qPCR |
| Agpat9 | forward primer, GGCCTTCGGATTATCCCTGG  reverse primer,  CTTGGGGGCTCCTTTCTGAA | qPCR |
| Dgat1 | forward primer, TCCGTCCAGGGTGGTAGTG  reverse primer,  TGAACAAAGAATCTTGCAGACGA | qPCR |
| Dgat2 | forward primer, GCGCTACTTCCGAGACTACTT  reverse primer,  GGGCCTTATGCCAGGAAACT | qPCR |
| Pparg | forward primer, CTCCAAGAATACCAAAGTGCGA  reverse primer,  GCCTGATGCTTTATCCCCACA | qPCR |
| Mouse-Actb | forward primer,  CTAGTAACCGAGACAT  reverse primer,  GGACGCCACAGCGGCC | qPCR |
| Mouse-D-loop | forward primer, AGGCATGAAAGGACAGCA  reverse primer,  TTGGCATTAAGAGGAGGG | qPCR |
| Mouse-18sRNA | forward primer, GAGAAACGGCTACCACATCC  reverse primer,  CACCAGACTTGCCCTCCA | qPCR |

**Table S5. List of primary antibodies**

| **Name** | **Source** | **Catalog** | **Application** | **Dilution** |
| --- | --- | --- | --- | --- |
| Anti-Rab1A antibody | Proteintech | 11671-1-AP | Western blot | 1:1000 |
|  |  |  | IF/IHC | 1:300 |
| Anti-LC3B antibody | Proteintech | 18725-1-AP | Western blot | 1:1000 |
|  |  |  | IF | 1:200 |
| Anti-TOM20 antibody | Proteintech | 66777-1-Ig | Western blot | 1:1000 |
|  |  |  | IF | 1:250 |
| Anti-phospho-p70 S6K(Thr389) antibody | CST | 9234 | Western blot | 1:1000 |
| Anti- p70 S6 Kinase antibody | CST | 2708 | Western blot | 1:1000 |
| Anti-phospho-Akt (Ser473) antibody | CST | 4060 | Western blot | 1:1000 |
| Anti-Akt(Pan) antibody | CST | 2920 | Western blot | 1:1000 |
| Anti-β-Actin antibody | CST | 8457 | Western blot | 1:1000 |
| Anti-PARK2/Parkin antibody | Proteintech | 14060-1-AP | Western blot | 1:1000 |
| Anti-PINK1 (phosphoT257) antibody | Abcam | ab303532 | Western blot | 1:1000 |
| Anti-PINK1 (phosphoS228) antibody | CST | 89010 | Western blot | 1:1000 |
| Anti-GOLGA2/GM130 antibody | Proteintech | 11308-1-AP | IF | 1:200 |
| Anti-COXIV antibody | Proteintech | 11242-1-AP | Western blot | 1:1000 |
| Anti-PINK1 antibody | Proteintech | 23274-1-AP | Western blot | 1:1000 |
| Anti-p-ERK1/2 (Thr202/Tyr204) antibody | Proteintech | 28733-1-AP | Western blot | 1:1000 |
|  |  |  | IF/IHC | 1:250 |
| Anti-p-p44/42 MAPK(Erk1/2) (Thr202/Tyr204) antibody | CST | 4370 | Western blot | 1:1000 |
| Anti-ERK1/2 antibody | Proteintech | 1257-1-AP | Western blot | 1:1000 |
| Anti-p-MEK1/2 (Ser217/221)antibody | CST | 9154 | Western blot | 1:1000 |
| Anti-MEK1/2 antibody | CST | 9126 | Western blot | 1:1000 |
| Anti-p-c-Raf (Ser338) antibody | CST | 9427 | Western blot | 1:1000 |
| Anti-c-Raf antibody | CST | 9422 | Western blot | 1:1000 |
| Anti-phospho-Raf1-S338 antibody | ABclonal | AP0498 | Western blot | 1:1000 |
|  |  |  | IF/IHC | 1:200 |
| Anti-phospho-A-Raf (Ser299) antibody | CST | 4431 | Western blot | 1:1000 |
| Anti- A-Raf antibody | CST | 4432 | Western blot | 1:1000 |
| Anti-HA-Tag antibody | CST | 3724 | Western blot | 1:1000 |
|  |  |  | IF/PLA | 1:200 |
| Anti-DDDDK-Tag antibody | ABclonal | AE005 | Western blot | 1:1000 |
|  |  |  | IF/PLA | 1:200 |
| Anti-RAF1 antibody | Abmart | MB0148S | IF/PLA | 1:200 |
| Anti-ATF4 antibody | Proteintech | 10835-1-AP | Western blot | 1:1000 |
| Anti-CHOP antibody | Proteintech | 15204-1-AP | Western blot | 1:1000 |
| Anti-GRP78/BIP antibody | Proteintech | 11587-1-AP | Western blot | 1:1000 |
| Anti-p-Acetyl-CoA Carboxylase (Ser79) antibody | CST | 11818 | Western blot | 1:1000 |
| Anti-Acetyl-CoA Carboxylase antibody | CST | 3676 | Western blot | 1:1000 |
| Anti-p-AMPKα (Thr172) antibody | CST | 2535 | Western blot | 1:1000 |
| anti-AMPKα antibody | CST | 5831 | Western blot | 1:1000 |
| Anti-p-AMPKβ1 (Ser182) antibody | CST | 4186 | Western blot | 1:1000 |
| Anti-AMPKβ1/2 antibody | CST | 4150 | Western blot | 1:1000 |
| Anti-p-JNK1/2 (Thr183/Tyr185) antibody | CST | 4668 | Western blot | 1:1000 |
| Anti-SAPK/JNK antibody | CST | 9252 | Western blot | 1:1000 |
| Anti-p-p38MAPK (Thr180/Tyr182) antibody | CST | 4511 | Western blot | 1:1000 |
| Anti-p38MAPK antibody | CST | 9212 | Western blot | 1:1000 |
| Anti-p-B-Raf-S445 antibody | ABclonal | AP0012 | Western blot | 1:1000 |
| Anti-B-Raf pAb antibody | ABclonal | A15033 | Western blot | 1:1000 |
| Anti-SREBF1 antibody | Proteintech | 14088-1-AP | Western blot | 1:1000 |
| Anti-FASN antibody | Proteintech | 10624-2-AP | Western blot | 1:1000 |
| Anti-SCD Polyclonal antibody | Proteintech | 28678-1-AP | Western blot | 1:1000 |
| Anti-ATGL antibody | CST | 2439 | Western blot | 1:1000 |
| Anti-PGK1 antibody | Proteintech | 17811-1-AP | IF/IHC | 1:250 |
| Anti-Phospho-Parkin (S65) antibody | CST | 36866 | Western blot | 1:1000 |
| Anti-GAPDH antibody | Proteintech | 60004-1-Ig | Western blot | 1:10000 |
| Anti-FIS1 antibody | Proteintech | 10956-1-AP | Western blot | 1:1000 |
| HRP-conjugated Goat Anti-Mouse IgG | Proteintech | SA00001-1 | Western blot | 1:10000 |
| Anti-OPA1 antibody | Proteintech | 27733-1-AP | Western blot | 1:1000 |
| Anti-MFN1 antibody | Proteintech | 13798-1-AP | Western blot | 1:1000 |
| Anti-rabbit IgG, HRP-linked Antibody | CST | 7074 | Western blot | 1:8000 |

CST: Cell Signaling Technology

IF: Immunofluorescence

IHC: Immunohistochemistry
